# *Plasmodium*-specific antibodies block in vivo parasite growth without clearing infected red blood cells

**DOI:** 10.1371/journal.ppat.1007599

**Published:** 2019-02-27

**Authors:** Jasmin Akter, David S. Khoury, Rosemary Aogo, Lianne I. M. Lansink, Arya SheelaNair, Bryce S. Thomas, Pawat Laohamonthonkul, Clara P. S. Pernold, Matthew W. A. Dixon, Megan S. F. Soon, Lily G. Fogg, Jessica A. Engel, Trish Elliott, Ismail Sebina, Kylie R. James, Deborah Cromer, Miles P. Davenport, Ashraful Haque

**Affiliations:** 1 QIMR Berghofer Medical Research Institute, Herston, Brisbane QLD, Australia; 2 Infection Analytics Program, Kirby Institute, UNSW Australia, Kensington NSW, Australia; 3 University of Melbourne, Department of Biochemistry and Molecular Biology, Melbourne, Victoria, Australia; Francis Crick Institute, UNITED KINGDOM

## Abstract

*Plasmodium* parasites invade and multiply inside red blood cells (RBC). Through a cycle of maturation, asexual replication, rupture and release of multiple infective merozoites, parasitised RBC (pRBC) can reach very high numbers *in vivo*, a process that correlates with disease severity in humans and experimental animals. Thus, controlling pRBC numbers can prevent or ameliorate malaria. In endemic regions, circulating parasite-specific antibodies associate with immunity to high parasitemia. Although *in vitro* assays reveal that protective antibodies could control pRBC via multiple mechanisms, *in vivo* assessment of antibody function remains challenging. Here, we employed two mouse models of antibody-mediated immunity to malaria, *P*. *yoelii* 17XNL and *P*. *chabaudi chabaudi* AS infection, to study infection-induced, parasite-specific antibody function *in vivo*. By tracking a single generation of pRBC, we tested the hypothesis that parasite-specific antibodies accelerate pRBC clearance. Though strongly protective against homologous re-challenge, parasite-specific IgG did not alter the rate of pRBC clearance, even in the presence of ongoing, systemic inflammation. Instead, antibodies prevented parasites progressing from one generation of RBC to the next. *In vivo* depletion studies using clodronate liposomes or cobra venom factor, suggested that optimal antibody function required splenic macrophages and dendritic cells, but not complement C3/C5-mediated killing. Finally, parasite-specific IgG bound poorly to the surface of pRBC, yet strongly to structures likely exposed by the rupture of mature schizonts. Thus, in our models of humoral immunity to malaria, infection-induced antibodies did not accelerate pRBC clearance, and instead co-operated with splenic phagocytes to block subsequent generations of pRBC.

## Author summary

Malaria occurs when *Plasmodium* parasites replicate inside red blood cells, with the number of parasitised cells (pRBC) correlating with disease severity. Antibodies are highly effective at controlling pRBC numbers in the bloodstream, and yet we know very little about how they function *in vivo*. Human *in vitro* studies predict that antibodies may function in a number of ways, including via phagocytes or different complement mechanisms. However, to date it has been challenging to explore how antibodies might control parasite numbers *in vivo*. Here, we have used a unique method in mice, where clearance and replication of a single cohort of pRBC was closely tracked in the presence of protective antibodies. Surprisingly, antibodies played no role whatsoever in accelerating the removal of pRBC. Instead, antibodies were highly effective at preventing parasites from progressing from one generation of pRBC to the next. This process partly depended on host phagocytes. However, we found no role for complement-mediated direct killing. Together, our *in vivo* data suggest in mouse models that naturally-acquired antibodies do not clear pRBC, and instead prevent transition from one red blood cell to the next.

## Introduction

Clinical symptoms of malaria occur during the erythrocytic phase of infection, when *Plasmodium* parasites mature and replicate asexually in red blood cells (RBC) [1]. A key feature of the asexual life-cycle in RBC is the capacity for rapid population growth, because each parasite can produce many daughter parasites (up to 32 daughter merozoites). The fold-increase in parasitised RBC (pRBC) from one cycle to the next is expressed by Parasite Multiplication Rate, PMR, a useful measure of parasite growth [2,3,4]. Theoretically, PMR can be influenced by host and parasite factors, such as how many merozoites are produced per replicating parasite, or how effectively the host clears parasites from the bloodstream. Since parasite biomass correlates strongly with disease severity in *P*. *falciparum*-infected humans [5] and in experimentally-infected mice [6], there can be serious consequences for an infected host over just a few cycles of infection. For example, if PMR = 16, four cycles of infection could theoretically result in a 65,536-fold (16^4^) increase in pRBC density. Therefore, it is important to minimise parasite replication to reduce the risk and severity of malaria.

Passive transfer of immune serum, firstly in rhesus monkeys [7], and later in children with malaria [8], was shown to reduce parasitemia and symptoms in the majority of recipients. This antibody-mediated control showed a slow decline in parasitemia with time, consistent with antibody-mediated “clearance” of pRBC. However, the rate of decay of pRBC appeared slow compared to that seen in drug treatment. In addition, because of the periodic life-cycle of the parasite, decays in parasite numbers might also occur by blocking parasite replication. Nevertheless, these passive transfer studies demonstrated that parasite-specific antibodies can control pRBC numbers effectively in humans and animal models, although precise mechanisms were not elucidated. Substantial effort has since been made to determine both the antigenic targets of protective antibodies [9,10], and secondly, the mechanisms by which they control pRBC numbers *in vivo* (reviewed in [9,11,12,13]). Broadly speaking, these antibodies exhibit two major specificities, either to *Plasmodium* proteins exported to the surface of pRBC, or to those expressed on the surface of merozoites [9]. Within either class, a number of possible *in vivo* mechanisms have been proposed and revealed in elegant *in vitro* assays. These include Growth Inhibition Assays (GIAs) [14,15], which assess if antibodies can reduce parasite growth in RBC over a few replication cycles; opsonic phagocytosis assays [16,17,18] and antibody-dependent cellular inhibition (ADCI) assays [19], which assess whether antibodies facilitate uptake and/or inhibition by phagocytes; and complement-fixing assays and killing assays [20,21], which determine how well antibodies facilitate C1q deposition and complement-dependent direct killing. A clear correlation between functional efficacy in GIAs and immunity to malaria is lacking [22,23,24,25]. However, in recent years, the capacity to mediate opsonic phagocytosis or C1q-fixation *in vitro*, mostly of merozoites, has been shown to correlate with protection against *P*. *falciparum* malaria symptoms and high density parasitemia [18,20,21]. These reports suggested that antibody-mediated immunity to blood-stage infection can be mediated by binding to merozoites, but with necessary engagement of host factors including phagocytes and certain aspects of the complement system. More recently, it was also suggested that antibodies against the pRBC surface protein, PfEMP1, which drove opsonic phagocytosis *in vitro*, afforded partial protection against malaria, and that these antibodies were acquired slightly earlier in life than merozoite-targeting antibodies [26]. Thus, the epidemiological data in humans supports the concept that multiple co-existing mechanisms acting against merozoites and pRBC can contribute to antibody-mediated pRBC control during blood-stage infection. However, few if any of these mechanisms have been convincingly observed *in vivo*.

Here, we sought to explore *in vivo* mechanisms of action for protective antibodies, specifically those acquired via primary blood-stage infection. We employed our established RBC adoptive transfer system, which permits tracking of a single cohort of fluorescently-labelled pRBC, as well as direct estimation of PMR and its inhibition by antibodies. We recently reported this technique for *P*. *berghei* ANKA parasites, to study the effect of anti-malarial drugs and the innate immune system [27,28,29]. Here, we have employed our approach in two common, non-lethal blood-stage malaria mouse models, *P*. *yoelii* 17XNL and *P*. *chabaudi chabaudi* AS infections in C57BL/6J mice, in which antibody-mediated immunity to homologous re-challenge with either parasite is generated after a single, self-resolving or chronic primary infection[30,31,32]. Interestingly, like human-infective *Plasmodium* species, *P*. *yoelii* encodes hundreds of pRBC surface proteins, called *yirs*, and several merozoite proteins, all of which are targets of the host immune system *in vivo* [33,34,35]. Thus, using these two established *in vivo* models of antibody-mediated immunity, we sought to determine how infection-induced antibodies control pRBC numbers *in vivo*.

## Results

### Blood-stage *P*. *yoelii* 17XNL infection generates passively transferable immunity

Passive transfer of immunoglobulin from immune adults has previously been shown to reduce parasitemias in children with malaria [8], via mechanisms that remain to be elucidated *in vivo*. Here, we first examined the utility of a mouse model, *Py*17XNL infection of C57BL/6J mice, for studying antibody control of blood-stage parasites *in vivo*. We and others previously reported that in *Py*17XNL primary infection, parasitemia is largely resolved by 30 *dpi* and that *Py*17XNL-specific IgG levels subsequently peak around 60–70 *dpi* [36,37]. Firstly, we determined whether sera from mice that had resolved a primary *Py*17XNL infection, and which harboured parasite-specific IgG (S1A Fig), could restrict parasitemia when administered therapeutically to mice infected with homologous parasites (S1B Fig). At day 3 *p*.*i*. when parasitemia was patent (~0.05%), infected mice were transfused with a single dose of immune serum, or non-immune control serum. As expected, the majority (5/6) of those receiving immune serum controlled parasitemia over the next 36 hours more effectively than non-immune serum recipients (S1B Fig).

**Fig 1:**
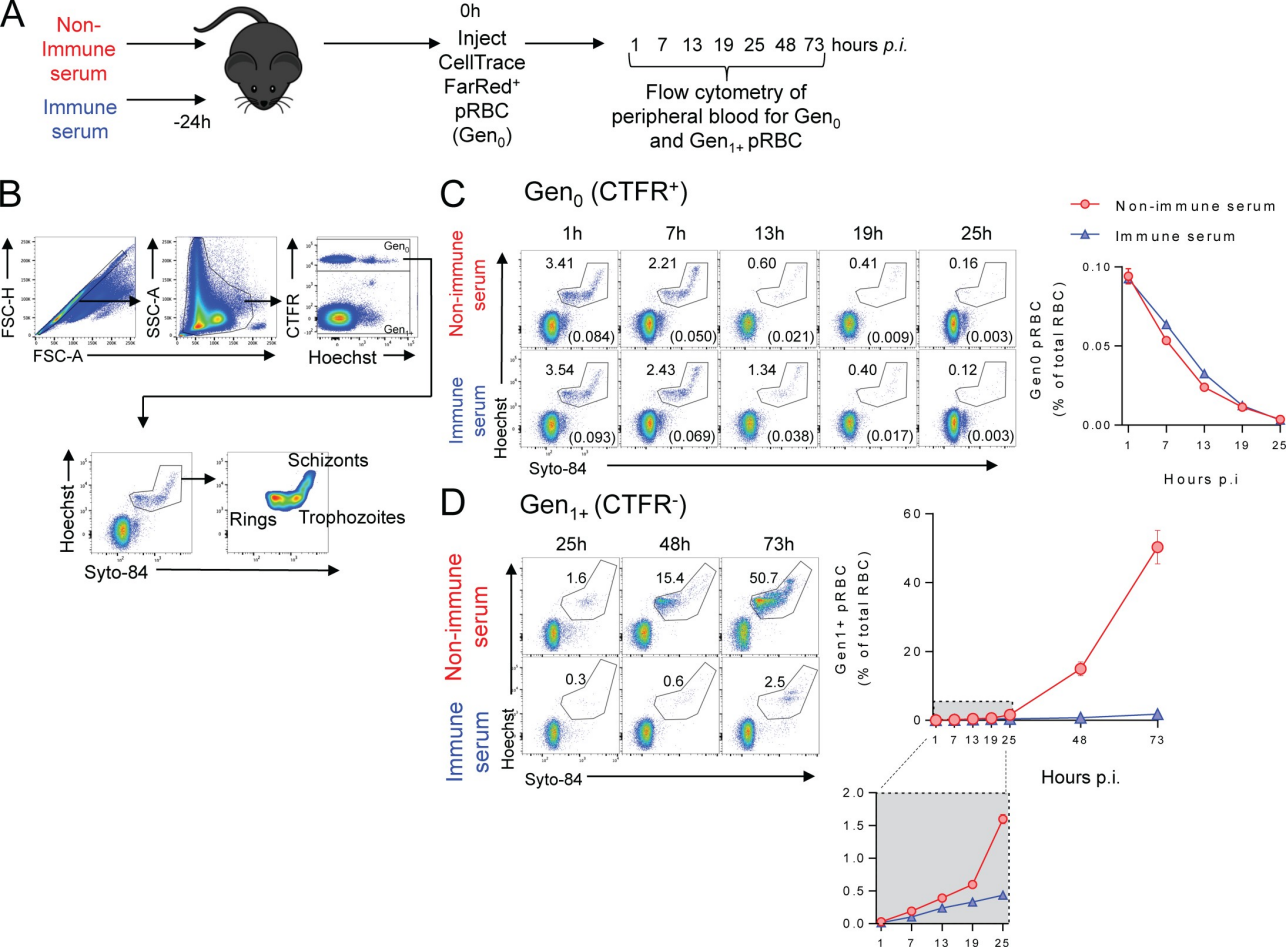
Infection-induced antibodies control *Py*17XNL parasite growth, not by accelerating pRBC clearance, but by blocking subsequent generations of pRBC. (A) Schematic showing that mice (n = 5/group) were injected with serum from *Py*17XNL-immune or non-immune mice 24h prior to challenge with CTFR-labelled *Py*17XNL-infected pRBCs and the progression of infection tracked by flow cytometry. (B) FACS gating strategy employed to analyse Gen_0_ and Gen_1+_ pRBC: forward scatter area and height (FSC-A and FSC-H) used to identify single cells, and side scatter area (SSC-A)/FSC-A used to identify RBCs. CTFR^+^ RBCs were the initial generation (Gen_0_), while Hoechst 33342/Syto 84 co-staining identified pRBCs and their life stages. (C) Representative FACS plots for time-course analysis of loss of Gen_0_ (CTFR^+^) pRBC, with numbers showing % of CTFR^+^ RBC containing parasites, also expressed in brackets and the summary graph as a % of total RBC in recipient mice. (D) Representative FACS plots for time-course analysis of Gen_1+_ (CTFR^-^) pRBC, and summary graph showing emergence of Gen_1+_ pRBCs over time. Data are representative of six independent experiments, each showing similar results.

Next, we hypothesized that antibodies elicited by *Py*17XNL infection could accelerate pRBC clearance by the host. To assess this, we used our established RBC adoptive transfer protocol [27,28,29]. Briefly, we harvested RBC from *Py*17XNL-infected WT passage mice (typically infected for 4-days, harbouring parasitemias of 2–10%, and exhibiting ~70% ring-stages), fluorescently-labelled them with CellTrace Far-Red (CTFR)(Fig 1A), and transferred this first generation, termed Gen_0_ (including un-infected, CTFR-labelled-RBC) into mice given *Py*17XNL-immune serum or non-immune control serum 24 hours previously (Fig 1A)—we note in this protocol that mice were challenged with high doses of pRBC (~10^7^ pRBC/mouse). We first assessed “total” parasitemia (ie. disregarding CTFR-staining of RBCs), since this is a more conventional assessment of pRBC in experimental animals or humans (S2A Fig). This confirmed that immune serum controlled total parasitemia over the first 3 days of high-dose homologous challenge compared to controls (S2A Fig). Thus, as expected, primary *Py*17XNL infection had elicited immunity that could be passively transferred under both therapeutic and high-dose challenge conditions *in vivo*.

### Infection-induced, *Py*17XNL-specific IgG antibodies do not accelerate pRBC clearance, but prevent parasites progressing to the next generation of RBC

Next, we made use of CTFR-labelling on pRBC to specifically study the initial generation (Gen_0_) of pRBC over the first 25 hours post-challenge (Fig 1B and 1C & S3 Fig). We used Hoechst33342 and Syto84 nucleic acid dyes to identify parasite life-cycle stages of Gen_0_ pRBC over the first 25 hours. Similar to our previous reports using *P*. *berghei* ANKA [27,28,29], Gen_0_ *Py*17XNL parasites matured into schizonts (S3 Fig) and were lost from circulation, mostly likely due to a combination of maturation-induced rupture and pRBC clearance. We recently reported that host factors can slow parasite maturation *in vivo* [27]. However, Gen_0_ parasite maturation occurred equivalently in immune and non-immune serum recipients over the 25-hour period (S3 Fig). Surprisingly, however, the loss of Gen_0_ pRBC was also equivalent between immune and non-immune serum recipients (Fig 1C), suggesting that pRBC clearance had been unaffected. Thus, our data reveal that immune serum, though protective against increases in parasitemia, did not influence the rate of pRBC clearance by the host.

We next assessed whether antibodies impeded Gen_0_ parasites from successfully infecting subsequent generations of un-infected RBCs (termed Gen_1+_), which we detected through the presence of parasites in CTFR^neg^ RBC (Fig 1B). While we observed rapid appearance of Gen_1+_ pRBC in non-immune serum recipients, immune serum substantially blocked the emergence of Gen_1+_ pRBC (Fig 1D). The PMR (the average number of Gen_1_ pRBC generated at 25h from each transferred Gen_0_ pRBC at 1h) was reduced to 3.7±0.1 by immune serum, compared to 11.5±0.8 in non-immune serum recipients, indicating that passively transferred immune serum prevented transit from one generation of RBC to the next.

However, given that donor Gen_0_ pRBC had been generated in WT passage mice, it is possible that natural antibodies or rapidly-generated IgM produced in the passage mice might coat donor pRBC prior to harvest and transfer, and thus interfere with the function of antibodies in immune serum. To test this, we repeated the above experiment using Gen_0_ donor pRBC generated either in *rag1*^*-/-*^ passage mice that completely lack antibody responses, or WT passage controls (S4A Fig). Both *rag1*^*-/-*^ and WT-generated Gen_0_ pRBC were cleared similarly in immune and non-immune serum recipients (S4 Fig), suggesting prior antibody coating of our donor pRBC was not a confounding factor in our studies.

Next, to confirm that infection-induced IgG was responsible for the passive transfer of protection, we repeated above experiments with purified IgG from immune or control serum (S5A Fig), alongside non-purified serum (S5B and S5C Fig). We found that purified IgG from immune serum did not alter the rate of Gen_0_ pRBC clearance (S5B Fig), but once again prevented emergence of Gen_1+_ pRBC in a similar manner to non-purified immune serum (S5C Fig). Together, our data confirmed that parasite-specific IgG controlled infection, not via pRBC clearance or impaired parasite maturation, but by blocking the progeny of Gen_0_ pRBC from establishing subsequent generations of pRBC.

### Neither cellular immunity nor systemic inflammation cause antibodies to clear pRBC

**Fig 2:**
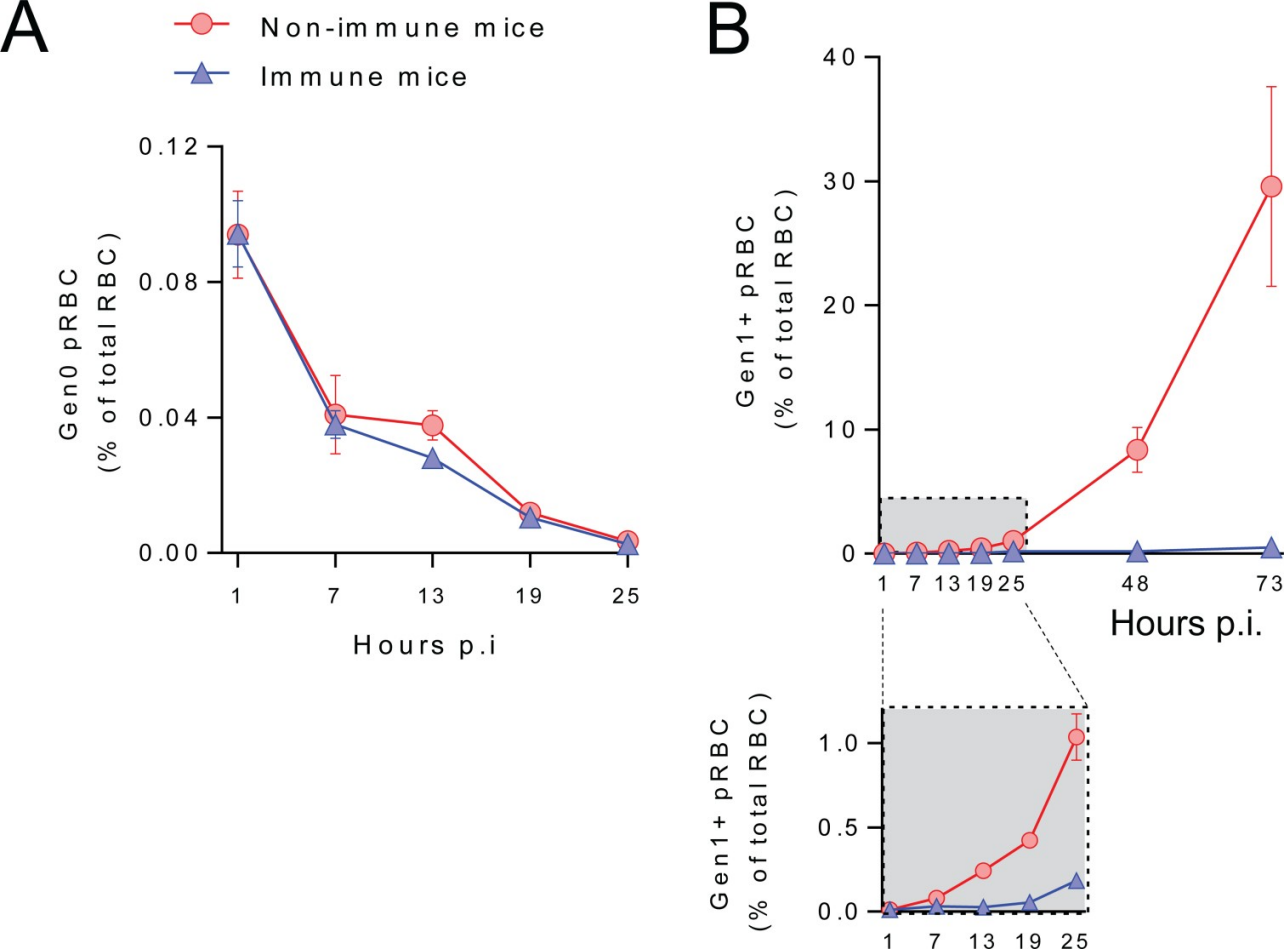
Combined cellular and humoral immunity do not accelerate pRBC clearance. *Py*17XNL-immune and non-immune mice (n = 5/group) were transferred with CTFR^+^ *Py*17XNL-infected pRBC, with peripheral RBC monitored at timepoints indicated for: (A) loss of Gen_0_ (CTFR^+^) pRBC over the first 25 hours, and (B) emergence over 3 days of Gen_1+_ (CTFR^-^) pRBC, with zoomed-in box showing the first 25 hours in more detail. All data representative of two independent experiments showing similar results.

The experiments described above involved passive transfer into naïve mice. We hypothesized that the lack of accelerated pRBC removal might be due to sub-optimal levels of antibodies transferred (in 200μl volumes). However, neither doubling the volume of immune serum (400μl), nor passive serum transfer closer to the time of challenge had any effect on pRBC removal, despite again restricting the emergence of Gen_1+_ pRBC (S6 Fig). It was also possible that additional immune mechanisms present in immune mice might be required to induce pRBC clearance by antibodies. To test this, we transferred Gen_0_ pRBC directly into immune mice (that had resolved a primary infection). As before, we observed no changes in Gen_0_ pRBC loss in immune mice compared to controls (Fig 2A), while total parasitemia (S2B Fig), and emergence of Gen_1_ pRBC was blocked, with PMR substantially reduced from 17.3±7.7 in non-immune mice to 0.78±0.29 in immune mice. These data suggested that the combined cellular and humoral components of immunity present in *Py*17XNL immune mice did not affect the rate of clearance of pRBC.

**Fig 3:**
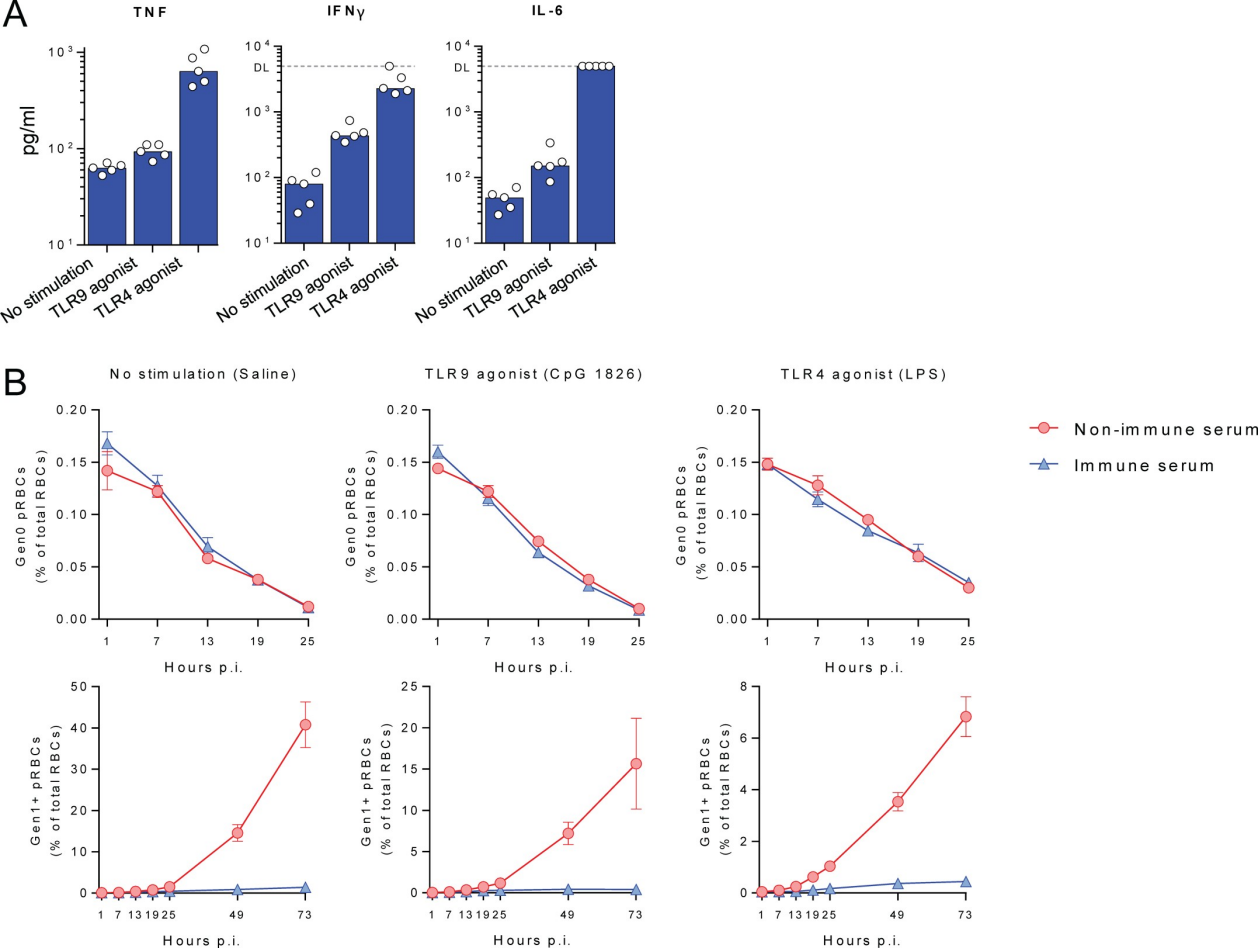
Systemic inflammation does not accelerate pRBC clearance by *Py*17XNL-specific antibodies. (A) Serum cytokine levels in mice (n = 5/group) receiving *Py*17XNL-immune serum and TLR4 (LPS) or TLR9 (CpG) agonists, measured at 7 hours after infection with CTFR^+^ pRBC. Each dot represents an individual mouse; DL = detection limit of assay. (B) Loss of Gen_0_ (CTFR^+^) pRBC over the first 25 hours, and emergence over 3 days of Gen_1+_ (CTFR^-^) pRBC in mice (n = 5/group) receiving *Py*17XNL-immune or non-immune serum, and stimulated via TLR4 or TLR9, or with control saline. Data representative of two independent experiments showing similar results.

Finally, our experiments to date had examined antibody function in the absence of ongoing inflammation, where factors such as systemic pro-inflammatory cytokines might be expected to augment antibody function. To examine infection-induced antibodies in this context, we transferred immune and non-immune serum into mice stimulated with TLR4 or TLR9 agonists, since *Plasmodium* infection can stimulate the innate immune system and pro-inflammatory cytokine production via these receptors (Fig 3) [38,39,40]. Despite clear upregulation of systemic cytokines IFNγ, TNF and IL-6 (Fig 3A), neither TLR agonist induced antibodies to accelerate the clearance of Gen_0_ pRBC (Fig 3B). Together our data indicated that the failure of infection-induced antibodies to accelerate pRBC clearance was unlikely due to the lack of accompanying cellular immunological memory or ongoing systemic inflammation.

### *Py*17XNL-specific antibodies require splenic macrophages and dendritic cells, but not complement C3/C5-mediated killing for optimal function *in vivo*

**Fig 4:**
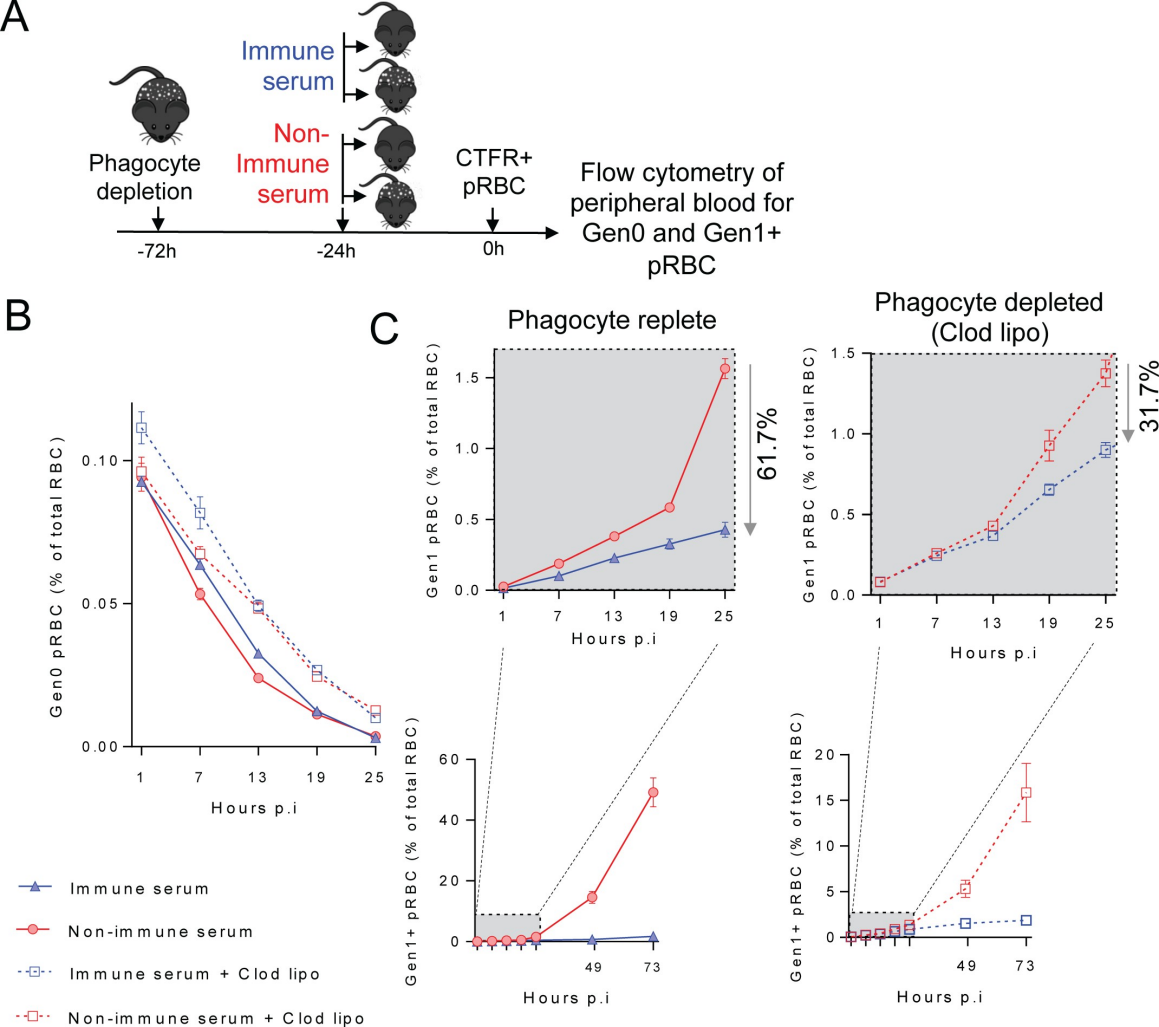
*Py*17XNL-specific antibodies require phagocytic cells for optimal *in vivo* function. (A) Schematic showing that 4 groups of mice (n = 5/group) were treated with clodronate liposomes or left untreated, and then given immune or non-immune control serum prior to infection with CTFR^+^ *Py*17XNL-infected pRBC, with peripheral blood assessed at time points indicated for: (B) loss of Gen_0_ (CTFR^+^) pRBC and (C) emergence over the first 25 hours of Gen_1+_ (CTFR^-^) pRBC in phagocyte replete, or clodronate liposome-treated mice receiving immune or non-immune serum. %’s indicate the reduction in PMR elicited by immune serum compared with non-immune control serum, with later timepoints beyond 25 hours also shown. Data representative of three independent experiments showing similar results.

We next hypothesised that additional host factors might be required for infection-induced antibodies to function *in vivo*. *In vitro* experiments have implicated phagocytes in antibody-mediated immunity to malaria in humans, via opsonic phagocytosis and antibody-dependent cellular inhibition (ADCI). To assess *in vivo* roles for phagocytes, we administered clodronate liposomes two days prior to transfer of immune or non-immune control serum. As expected, clodronate substantially depleted F4/80^+^ macrophages and CD11c^hi^ conventional dendritic cells in the spleen, with only minor effects on other cells such as monocytes and neutrophils (S7A and S7B Fig). We then transferred and tracked CTFR^+^ (Gen_0_) pRBC as above (Fig 4A). Over the first 25 hours, clodronate-treatment modestly reduced the rate of clearance of Gen_0_ pRBC in mice receiving either immune or non-immune serum (Fig 4B). More importantly, however, clodronate treatment reduced antibody effectiveness, since immune serum reduced PMR by 61.7% (range: 50–70) in phagocyte-replete mice, yet by only 31.7% (range: 25–40) in mice depleted of splenic macrophages/dendritic cells (Fig 4C: P<0.0096, two-way ANOVA, significant interaction between serum administration and clodronate treatment, 5 mice/group, three independent experiments). Thus *Py*17XNL-immune serum was ~50% less effective in the absence of phagocytes over the first 25 hours. Nevertheless, at days 2–3 post-transfer, immune serum limited the emergence of later generations (Gen_1+_) of pRBC in both phagocyte-depleted and intact mice (Fig 4C), suggesting that phagocyte-independent antibody-mediated mechanisms might exist in our model systems. In summary, parasite-specific antibodies co-operated with splenic macrophages and dendritic cells to optimally restrict parasites transitioning from Gen_0_ to Gen_1_ RBC.

Finally, we also considered phagocyte-independent antibody mechanisms. Previous studies reported two mechanisms, complement-dependent merozoite invasion blockade requiring C1q-deposition, and complement-mediated direct lysis of merozoites via formation of C3/C5-dependent membrane attack complexes (MAC). To test for the importance of MAC formation in antibody function, we depleted mice of complement proteins C3/C5 (S8A and S8B Fig), using non-toxic, cobra venom factor (CVF) prior to transfer of immune or non-immune serum. Importantly, CVF-treatment does not target C1q. Having administered Gen_0_ pRBC we noted that as before, loss of Gen_0_ pRBC was equivalent amongst all groups (S8C Fig). We then compared PMR reduction elicited by *Py*17XNL-immune serum (compared to non-immune serum) in mice either replete or CVF-treated. We found no evidence that immune serum failed to control PMR in CVF-treated mice compared to saline controls (S8D Fig), with no significant effect observed in two experiments (P>0.12, two-way ANOVA interaction between immune serum administration and CVF treatment, 5 mice/group, 2 independent experiments). Therefore our data revealed no role for complement C3/C5-mediated killing in antibody function. Taken together, our data suggested that infection-induced antibodies co-operated with splenic macrophages and dendritic cells, but did not require complement-mediated direct killing for their function *in vivo*.

### Infection-induced *Py*17XNL-specific IgG binds not to the surface of intact pRBC, but to internal structures within schizonts

**Fig 5:**
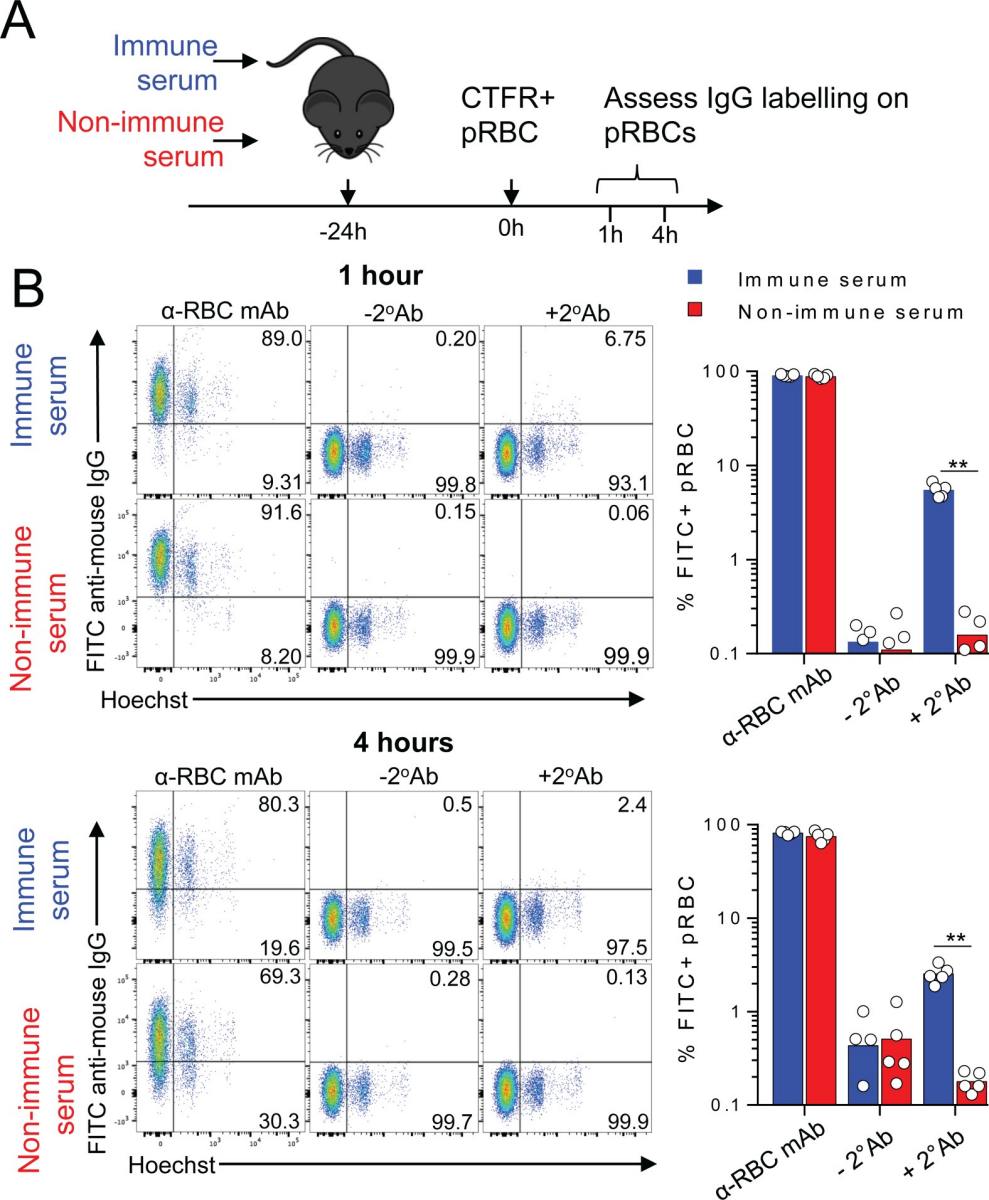
*Py*17XNL-specific IgG binds weakly to the surface of pRBC *in vivo*. (A) Schematic showing that mice (n = 5/group) were injected with immune or non-immune control serum 24h prior to infection with CTFR^+^ *Py*17XNL-infected pRBCs, with surface deposition of IgG on pRBC assessed 1h and 4h afterwards. (B) Representative FACS plots showing detection of *in vivo*-deposited mouse IgG on the surface of CTFR^+^ RBC (after 1h & 4h *in vivo* exposure), with positive controls stained *in vitro* with RBC-specific mouse IgG (34-3c). Data are representative of two independent experiments showing similar results. Statistics: Mann-Whitney t-test, **P < 0.01. (C-D).

Given that infection-induced IgG blocked parasite transit between RBC, but did not accelerate pRBC clearance, we hypothesized that antibodies in immune serum primarily target merozoites/internal structures that are exposed upon schizont rupture, rather than antigens on the surface of the infected RBC. To test this, we injected immune serum or non-immune control serum into mice followed by transfer of Gen_0_ pRBC (Fig 5A). After 1 or 4 hours in ciruclation to afford time for *in vivo* binding, we then removed and examined Gen_0_ pRBC for evidence of cell-surface binding of mouse IgG (Fig 5B). As a positive control for mouse IgG-deposition on RBC, control samples were incubated *in vitro* with a mouse monoclonal IgG (clone 34-3C) specific for murine RBC (Fig 5B). After 1 or 4 hours of *in vivo* exposure to immune serum, only 2–7% of pRBC bound mouse IgG, the extent of which was weak compared to 34-3C-treated controls (Fig 5B). Thus, consistent with our hypothesis, *Py*17XNL-specific IgG bound only weakly to the surface of pRBC *in vivo*.

**Fig 6:**
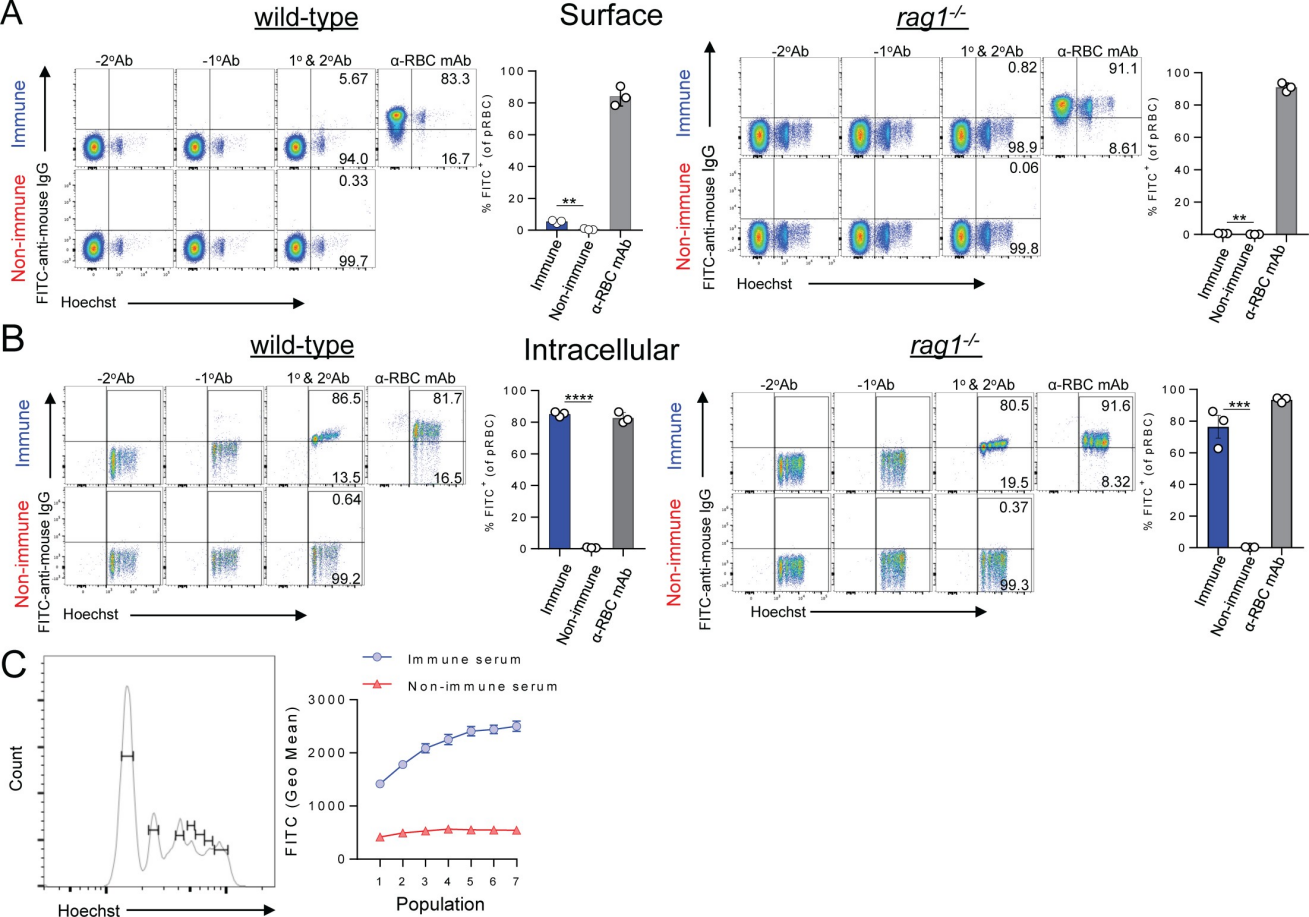
Infection-induced *Py*17XNL-specific IgG binds poorly to intact pRBC, but strongly to permeabilised schizonts. Detection of *in vitro* deposited IgG from immune or non-immune control serum either (A) on the surface of RBC, or (B) inside RBC from *Py*17XNL-infected wild-type mice and *rag1*^*-/-*^ passage mice after fixation/permeabilisation. Graph shows percentage of pRBC binding to mouse IgG after fixation/permeabilisation. (C) Representative histogram showing gating for identifying *Py*17XNL pRBC from wild-type mice containing different numbers of merozoites using Hoechst. Graph shows variation in geometric mean fluorescence intensity of *in vitro* IgG deposition inside fixed and permeabilised pRBC according to DNA content, when incubated with immune or non-immune control serum (assessed in triplicate); two-tailed Students T-test, p = 0.0004. Data in (A-C) are representative of six independent experiments for pRBC from WT mice, and two independent experiments using *rag1*^*-/-*^ passage mice.

**Fig 7:**
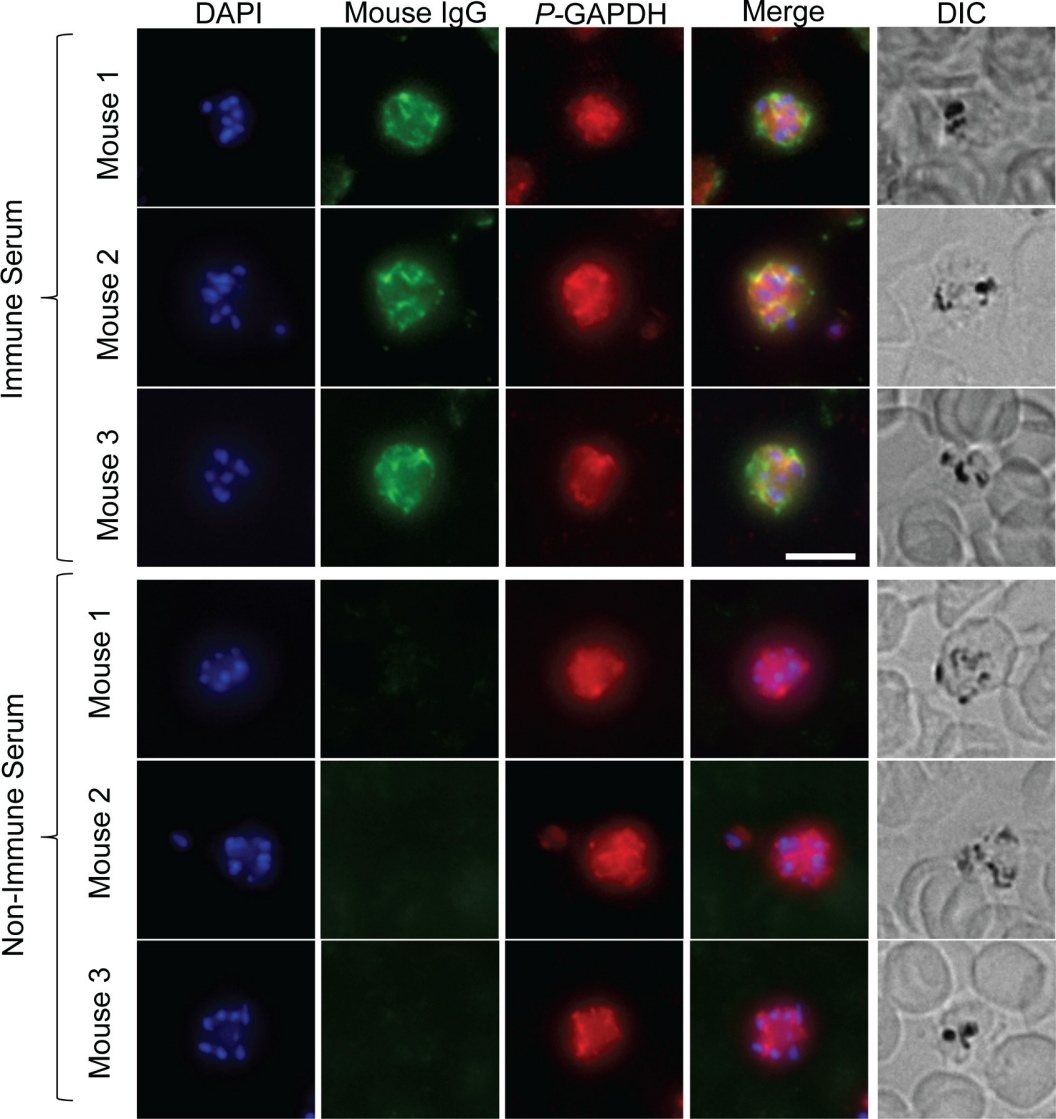
*Py*17XNL-specific IgG binds strongly to internal structures within schizonts of pRBC. *P*. *yoelii* 17XNL-infected blood was harvested from 3 separate *rag1*^*-/-*^ passage mice and stained with immune (top panels) or non-immune (bottom panels) serum (green). Parasites were counterstained with anti-GAPDH (red) to delineate the parasite, and DAPI (blue) to highlight the parasite nuclei. Merge images and DIC images are also shown. Images are representative of multiple pRBC assessed for each mouse. Scale Bar = 5**μ**m.

Next, to test whether infection-induced parasite-specific IgG could potentially bind internal parasite structures exposed during schizont rupture, we fixed and permeabilised pRBC (from either WT or *rag1*^*-/-*^ passage mice) *in vitro* prior to exposure to immune serum (Fig 6). Interestingly, we note that while fixation and permeabilisation lysed all the un-infected RBC in our preparation and perhaps early-ring stages (Fig 6A compared to 6B), later-stage pRBC remained intact, permitting immune-staining and flow cytometric assessment (Fig 6B). In stark contrast to pRBC-surface assessment (Fig 6A), ~90% of intact, permeabilised pRBC strongly bound IgG from immune serum, compared to <5% for control serum (Fig 6B). Furthermore, pRBC with the highest DNA content, indicative of progression through schizogony, exhibited the strongest IgG staining (Fig 6C). Finally, microscopy assessment revealed immune IgG binding to pRBC, particularly to multiple internal structures within schizonts, including but not limited to nascent, DAPI^+^ merozoites (Fig 7). Taken together, our data indicate that infection-acquired IgG antibodies bind poorly to the surface of pRBC, yet strongly to internal structures of late-stage pRBC, which may become exposed to antibodies *in vivo* upon schizont rupture.

### *P*. *chabaudi-*specific antibodies also protect without accelerating pRBC clearance

**Fig 8:**
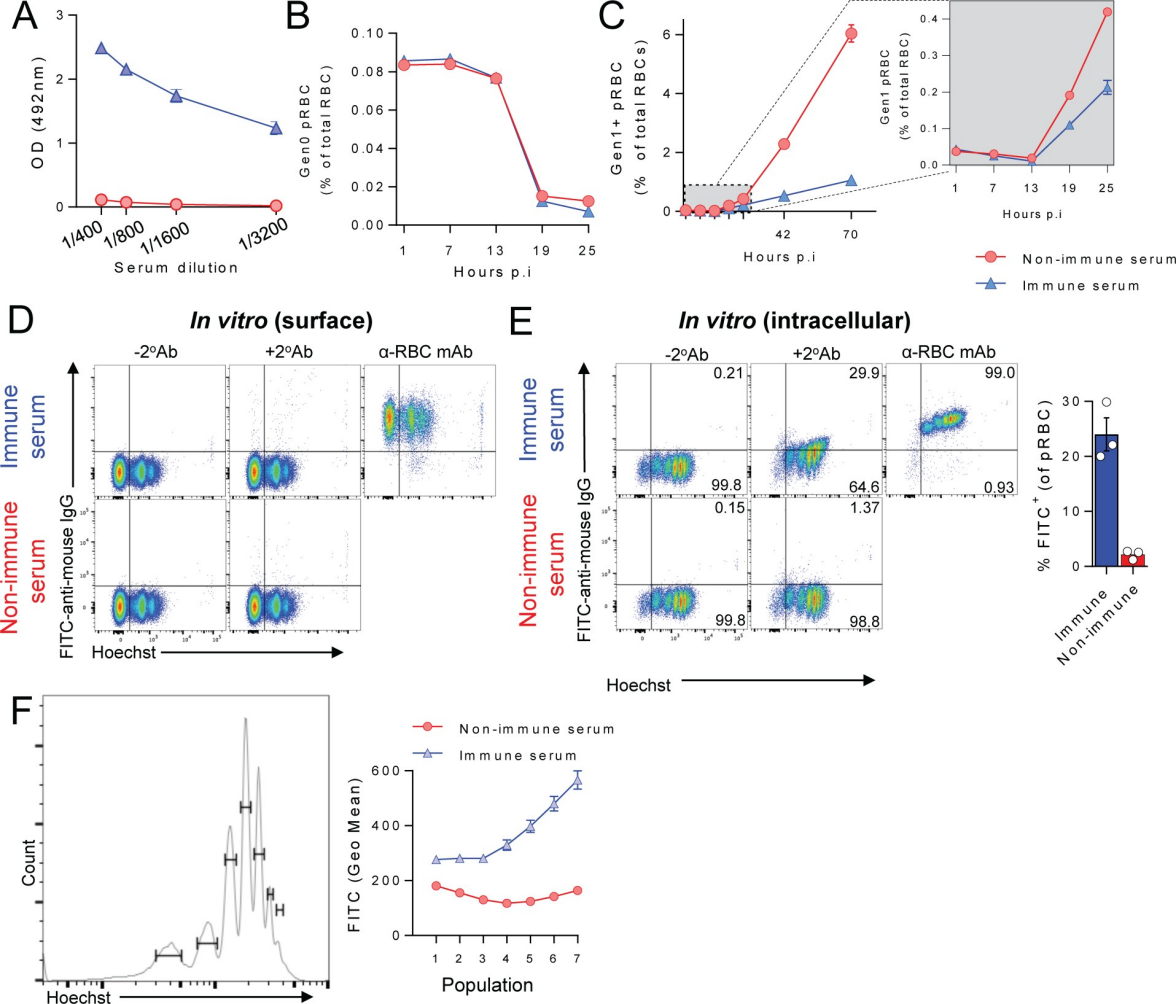
Antibodies induced by *P*. *chabaudi chabaudi* AS infection, also protect by blocking parasite transit between RBC, not accelerating pRBC clearance. (A) Assessment of *Pc*AS-specific total IgG in diluted sera from infected or age-matched naïve mice (n = 10/group), taken at day 40 *p*.*i*. with *Pc*AS. (B-C). Mice (n = 5/group) were injected with *Pc*AS-immune serum or non-immune control serum 24h prior to challenge with CTFR^+^ *Pc*AS-infected pRBCs, with peripheral blood monitored at times indicated for: (B) loss of Gen_0_ (CTFR^+^) pRBC over the first 25h, and (C) emergence of Gen_1+_ pRBC over 73h, with the zoomed-in box showing the first 25h in more detail. (D-E) Assessment of *in vitro* deposition of mouse IgG from *Pc*AS-immune or non-immune control serum performed in triplicate, either (D) on the surface of RBC from *Pc*AS-infected mice, or (E) inside RBC from *Pc*AS-infected mice after fixation/permeabilisation. Graph shows percentage of pRBC binding to mouse IgG after fixation/permeabilisation. (F) Representative histogram shows gating for identifying *Pc*AS-infected pRBC containing different amounts of parasite DNA using Hoechst. Graph shows variation in geometric mean fluorescence intensity of *in vitro* IgG deposition inside fixed and permeabilised pRBC according to DNA content, when incubated with immune or non-immune control serum (assessed in triplicate). Data in (D-F) are representative of two independent experiments each with similar results.

We next sought to determine whether similar antibody mechanisms act in a second model, *P*. *chabaudi chabaudi* AS infection. We harvested immune serum from *Pc*AS-infected wild-type C57BL/6J mice at 40 *dpi*, the peak of parasite-specific IgG in this model [36], and confirmed the presence of *Pc*AS-specific IgG (Fig 8A). Next, we employed the same approaches as above, transferring *Pc*AS-infected CTFR^+^ pRBC into naïve mice given *Pc*AS-immune serum or control serum (Fig 8B & 8C). The loss of Gen_0_ pRBC was again not influenced by immune serum (Fig 8B), although the emergence of Gen_1+_ pRBC was strongly blocked (Fig 8C), with PMR reductions of ~50% for immune serum compared to controls. Consistent with no effect on pRBC clearance, yet strong effects on Gen_0_ to Gen_1_ transition, we noted that *Pc*AS-specific IgG did not bind to the surface of pRBC *in vitro* (Fig 8D), but rather to intracellular structures inside late-stage, permeabilised pRBC (Fig 8E & 8F). Thus, in two independent models, infection-induced antibodies did not bind to molecules on the surface of pRBC, and protected by blocking parasite transit between RBCs.

## Discussion

Several mechanisms have been proposed to explain how *Plasmodium*-specific antibodies acquired through natural exposure may prevent or ameliorate malaria. Broadly speaking, these include three classes of mechanism: where antibodies act mostly alone, without other host processes required; where they serve as triggers for phagocyte-dependent processes (opsonic phagocytosis and antibody-dependent cellular inhibition (ADCI)); or where they activate complement-mediated direct killing. All three classes of mechanism could theoretically act on pRBC or free merozoites. However, it has been challenging to explore the importance of these *in vivo*.

Here, we utilised two of the most common *in vivo* models of humoral immunity to malaria, *P*. *yoelii* 17XNL and *Pc*AS infection of C57BL/6J mice, to dissect how infection-induced antibodies control parasite numbers during secondary challenge. This was combined with an RBC adoptive transfer technique to study parasite clearance and replication *in vivo* [27,28,29]. We provided evidence that effective control of pRBC numbers was mediated not by targeting pRBC for clearance by the host, either in naïve mice, or when cellular components of adaptive immune memory or ongoing systemic inflammation were added. Moreover, we provide evidence that splenic macrophages and dendritic cells are necessary for optimal *in vivo* antibody function, while complement C3/C5-mediated killing played no discernible role. Moreover, while infection-induced IgG antibodies bound very poorly to the surface of intact pRBC in either model, they bound well to internal structures of schizonts. Our flow cytometric and microscopy data suggest merozoites and other internal structures could be targets for antibodies once schizont rupture has occurred. Thus, we propose here a model in which infection-induced antibodies target merozoites and internal parasite structures exposed upon schizont rupture, and co-operate with splenic phagocytes to impair transit of parasites from one RBC to another.

Although our study indicated a role for splenic phagocytes in preventing parasites transitioning from one generation of RBC to the next, which of the two main phagocyte-dependent mechanisms, opsonic phagocytosis or ADCI, were involved remains to be determined. Given that ADCI is mediated *in vitro* by a monocyte-derived cytokine, Tumor Necrosis Factor (TNF) [19], future studies should explore an *in vivo* requirement for TNF in our system. Another important question arising from the reduction in antibody-efficacy after phagocyte depletion, is whether the partial effect was due to incomplete phagocyte depletion by clodronate liposomes, or indicates the existence of multiple mechanisms of antibody action. Although a single-dose of clodronate liposomes does not completely deplete phagocytes for more than a few days, it nonetheless was a very efficient way to remove splenic macrophages in particular. For that reason, we believe that other mechanisms likely contribute to parasite control in our models, such as blockade of merozoite invasion or pro-inflammatory responses to antibody-coated fragments of ruptured schizonts [20]. A further possibility is that non-phagocytic cells expressing Fc receptors may also mediate antibody-dependent control in our *in vivo* models. For example, a recent study demonstrated human NK cells mediated antibody-dependent cellular cytotoxicity against *Plasmodium falciparum-*infected RBC *in vitro* [41]. Future experiments and technological advances may be required to assess these mechanisms *in vivo* since it is not trivial to reliably visualise pRBC, merozoites, or schizont fragments interacting with phagocytes or other immune cells *in vivo*.

A recent study revealed in human samples that the capacity of merozoite-specific antibodies to facilitate deposition of the polymeric complement C1q complex strongly correlated with protection from high density parasitemia [20]. Moreover, *in vitro* assays indicated that although C1q deposition could increase direct complement-mediated lysis of merozoites, C1q deposition alone, ie. with no other components of the complement system present, was sufficient to enable antibodies to block merozoite invasion of RBC. Thus a conclusion from this paper was that merozoite-specific antibodies from children protected against high density parasitemia probably acted by blocking invasion, not by direct complement-mediated lysis of merozoites. Our study in mice is consistent with infection-acquired IgG blocking merozoite transit to the next generation of RBC, with no major role for complement-mediated direct lysis. However, in our study, we did not directly assess a role for antibody-mediated C1q deposition. Thus, it may be important in the future to examine whether parasite-specific antibodies are impaired in their capacity to block Gen_0_ to Gen_1_ transit in C1q^-/-^ mice. Another important question is whether the complement system is adequately functional in the C57BL/6J genetic background employed in this study, for any similarities to be drawn to human immunity to malaria. A previous assessment of complement in numerous inbred mouse strains focussed on the capacity to directly lyse cellular targets [42]. This report indicated that strains with lower complement lysis capabilities tended to have reduced amounts of C3, C5, C6 and C7 in their serum compared to strains with better complement lysis capacity, but similar levels of C1 proteins [42]. Given these findings, it is perhaps unsurprising that direct complement mediated lysis was not an important mechanism in our system for controlling infection. However, given that levels of C1 proteins are likely equivalent amongst most mouse strains [42], it remains to be determined whether C1q deposition contributes to antibody function in the *Py*17XNL and *Pc*AS systems. We hypothesise this to be the case since a previous report showed that genetic C1q deficiency in mice impaired immunity to re-challenge with blood-stage *Plasmodium* parasites [43].

Many, but not all, of the suggested mechanisms by which antibodies could serve to control pRBC numbers *in vivo* can be explored during *Py*17XNL infection in mice. For example, it has been suggested that antibodies could prevent cytoadherence, and thus sequestration, of pRBC to endothelial surfaces in various tissues [11,44]. Similarly, antibodies could prevent adherence of RBC to each other, also known as rosetting [11,44]. Neither of these two phenomena are well-established in the *Py*17XNL model. Therefore, our data cannot be taken as complete assessment of all the antibody mechanisms that prevent malaria in humans. Instead, our study emphasises that antibody binding to the pRBC surface is not necessary for control, while targeting free merozoites and/or ruptured schizonts appears to predominate in two of the most common mouse models of antibody immunity. It has previously been shown that *Py*17XNL parasite export variant surface proteins, *yirs*, to the surface of RBC during *in vivo* infection of mice. Moreover, *yirs* are clearly detected by the mouse immune system, since their variation is dependent upon the presence of adaptive immunity. In our study, we found evidence that intact schizont stage pRBC did bind parasite-specific IgG. It is possible that *yir* proteins could be the target for such antibodies, in a similar way that *Pf*EMP1 appears to be a major target for pRBC binding antibodies in human samples [26]. However, an important question from our study is why the IgG response to infection is dominated by antibodies specific to the contents of permeabilised schizonts, most likely merozoites and proteins released into circulation during rupture, and not to pRBC surface proteins. The answer to this question remains unclear at present but could be related to the ability of antigen-presenting cells to access and process pRBC surface proteins compared to merozoite proteins and the cellular debris released by pRBC rupture.

We previously described in our models, the kinetics with which parasite-specific IgM and IgG isotypes accumulate in mice infected with *P*. *chabaudi* and *P*.*yoelii* 17XNL [36,37]. However, this study was designed primarily to assess peak levels of parasite-specific IgG (at 40–60 days post-infection). Therefore, our experiments do not shed light on how the sequential generation of IgM and IgG classes might mediate immunity, nor how different immune cell-types might interface with these Ig classes. Future experiments could explore the functional roles of IgM and IgG sub-classes *in vivo*.

In this study, the serum transfer achieved only a fraction of the antibody concentration present in the serum of an immune mouse, yet elicited clear protection even against high inocula. Mice were therefore administered sufficient antibodies to protect against large infectious doses. However, this model appears different to natural *P*. *falciparum* infection scenarios, where protective antibody levels might be relatively low, and where individuals are infected with much lower numbers of blood-stage parasites. Nevertheless, we speculate that antibody mechanisms outlined in our mouse studies may be relevant for understanding immunity in humans.

Our study also raises questions regarding how antibody-mediated immunity might be boosted in a rational manner. For example, could increasing the plasma concentration of antibodies that recognise pRBC further improve protection? This may indeed be the case. However, given that antigenic variation is a major feature of pRBC surface proteins, it will likely be challenging to induce pRBC-targeting antibodies that elicit long-lasting immunity to high-density parasitemia in humans. Nevertheless, in summary, our data using *in vivo* models suggest that pRBC clearance is not a crucial mechanism for naturally-acquired antibodies to control parasitemia. Instead, we propose that binding of affinity-matured IgG to the surface of merozoites and/or to fragments of ruptured schizonts protects against blood-stage *Plasmodium* infection via multiple mechanisms including those mediated by splenic macrophages and dendritic cells. Our pre-clinical *in vivo* data provide further insight into the types of antibody functions that might protect naturally-exposed and immunised humans against malaria.

## Materials and methods

### Ethics statement

Female C57BL/6J mice aged 6–12 weeks were purchased from the Australian Resource Centre (Canning Vale, Perth, WA, Australia) and maintained under conventional conditions. This study was carried out in strict accordance with guidelines from The National Health and Medical Research Council of Australia, as detailed in the document *Australian Code of Practice for the Care and Use of Animals for Scientific Purposes*, 7th edition, 2004. All animal procedures and protocols were approved (A1503-601M) and monitored by the QIMR Berghofer Medical Research Institute Animal Ethics Committee.

### Parasites and generation of immune mice

*P*. *yoelii* 17XNL (*Py*17XNL) or *P*. *chabaudi chabaudi* AS (*Pc*AS) parasites were used after defrosting frozen, infected stabilate blood and performing a single *in vivo* passage in wild-type (WT) or *rag1*^*-/-*^ C57BL/6J mice (Animal Resource Centre, Perth, Australia) until a parasitemia of 1–5% was reached. Immune mice (and thus, immune sera) were generated by infecting WT mice with 10^4^ *Py*17XNL-containing pRBC or 10^5^ *Pc*AS-containing pRBC, via intravenous (*i*.*v*.) injection using a lateral tail vein (200μl using 26G needles), and confirming firstly that infection had been initiated around day 7–14 days post-infection (*dpi*), and secondly that peripheral blood parasitemia had dropped below 0.1% by flow cytometry (described below) by 40–50 *dpi* for *Pc*AS, and 60–80 *dpi* for *Py*17XNL. Age-matched naïve control mice were housed in the animal facility to generate non-immune mice and control, non-immune serum. Unless stated otherwise, mice were administered 200μl of immune or control serum, or 500μg of purified IgG, via intravenous injection using a lateral tail vein.

### Adoptive transfer of fluorescently-labelled RBC

Adoptive transfer of a single cohort of fluorescently-labelled pRBC was carried out as previously described [27,28,29]. pRBC, including un-infected RBC, were collected from *Py*17XNL or *Pc*AS-infected passage mice by cardiac puncture. Heparinised donor blood was washed twice in Ca^2+^/Mg^2+^-free phosphate buffered saline (PBS-A), and stained in CellTrace Far Red (CTFR) according to manufacturer’s instructions. Briefly, 50μg CTFR was dissolved for ten minutes in 25μl tissue culture grade dimethyl sulphoxide. This was then added to 5ml of resuspended RBC in PBS-A. RBC were stained in the dark, at room temperature with constant rolling for 15 minutes, and then washed twice in 10x volumes of PBS-A. Successful CTFR-labelling of RBC was confirmed by flow cytometry using an LSRII Fortessa analyser (BD Biosciences) and FlowJo software (Treestar, CA, USA). CTFR-labelled RBC were resuspended in 2ml volumes per donor mouse, and injected in 200μl volumes via *i*.*v*. injection using 26G needles.

### Flow cytometric analysis of pRBC in peripheral blood

Our established flow cytometric method for *P*. *berghei* ANKA parasites, was employed here to track the first generation of injected *Py*17XNL or *Pc*AS-containing pRBC, termed Gen_0_ pRBC, and to distinguish these from subsequent generations of pRBC (termed Gen_1+_), which emerged as un-labelled, endogenous RBC became infected by the merozoite progeny of Gen_0_ pRBC. Finally, our staining approach also permitted assessment of parasite life-stages (ring, trophozoite and schizont), as previously reported [27,28,29]. Briefly, a single drop of blood from a tail bleed was diluted and mixed in 200 μl of RPMI medium containing 5 U/ml heparin sulphate. Diluted blood was simultaneously stained for 30 min in the dark at room temperature with cell-permeant RNA/DNA stain, Syto84 (5 μM; Life Technologies) and with DNA stain, Hoechst 33342 (10 μg/ml; Sigma). Staining was quenched with 10 volumes of ice-cold RPMI medium, and samples immediately analysed by flow cytometry using an LSRII Fortessa analyser (BD Biosciences) and FlowJo software (Treestar, CA, USA). FSC-Area (FSC-A) and FSC-Height (FSC-H) was used to exclude doublets (the result of two cells being detected simultaneously by the flow-cytometer). FSC-A and side scatter (SSC-A) was used to distinguish RBCs from debris and white blood cells on the basis of size and granularity. Infected RBCs were detected as co-expressing Hoechst 33342 and Syto84, with expression of either one alone being insufficient to identify the presence of parasites. Adoptively-transferred Gen_0_ pRBCs were readily distinguished from endogenous RBC, and importantly from Gen_1+_ pRBC, by CTFR-labelling.

### Purification of IgG from immune serum

Purification of IgG from immune serum or non-immune serum was performed using Protein G Sepharose4 Fast Flow (GE Healthcare) column, according to the manufacturers’ protocol. Briefly, the column was equilibrated with Running Buffer (0.02M sodium phosphate, 0.01% sodium azide, pH 7) prior to running serum (diluted 1:3 with Running Buffer) through at ~1.5 ml/minute. Flow-through (FT) was collected and prior to elution, the column was washed with Running Buffer to remove any unbound protein. IgG was eluted from the column with Elution Buffer (0.1M glycine, 0.01% sodium azide, pH 2.7) and the pH of the eluate was neutralised by addition of 1M Tris-HCl pH 9. Buffer exchange of the eluate to 0.9% saline was performed using Amicon Ultra-15 10K Centrifugal Filter devices (Merck Millipore) and sterilised using Acrodisc 0.2μm Mustang E Membrane Units (PALL Life Sciences). The concentration of purified IgG was determined using a NanoDrop ND-1000 and the IgG was then stored at -80°C until use.

For SDS-PAGE analysis, samples were resuspended in 4x NativePAGE Sample Buffer (ThermoFisher Scientific) and β-mercaptoethanol (10% final concentration) was added prior to denaturing at 95°C for 5 minutes. Samples were cooled on ice for 5 minutes and then separated by 10% SDS-PAGE. Protein sizes were estimated by loading of a BenchMark Pre-stained Protein Ladder (Life Technologies) and an anti-mouse CD3 rat IgG (Biolegend) was used as a control. The SDS-PAGE gel was stained with Coomassie Brilliant Blue R250 and imaged on an Odyssey CLx imaging system (LI-COR Biosciences).

### Preparation of crude parasite antigen

Crude antigen extract from *Py*17XNL-infected pRBC or *Pc*AS-infected pRBC was prepared using an adapted version of a previously described protocol [45]. Briefly, mice were infected as described above. When parasitemias reached 20–30%, blood was collected by cardiac puncture into heparinized tubes. RBC were washed once in RPMI at 1200rpm for seven minutes at room temperature, and then lysed using ultrapure water followed by four washes in ice-cold PBS at 16,000xg for 25 minutes at 4°C, as well as three cycles of freezing (two hours at -80°C) and thawing (30 minutes at room temperature). Extracts were also processed from RBCs of uninfected C57BL/6J mice, for use as negative controls in ELISA. The concentration of proteins in the purified extracts was determined by Bradford assay (Thermo Scientific). All extracts were stored at -80°C until use.

### Detection of parasite-specific serum antibodies by ELISA

Costar EIA/RIA 96-well flat bottom plates were coated overnight at 4°C with 2.5μg of soluble antigen/ml in bicarbonate coating buffer (pH9.6). Wells were washed three times (all washes in 0.005% Tween in PBS) and then blocked for 1hr at 37°C with 1% BSA in PBS. Wells were washed three times, 100ul of sera diluted 1/400, 1/800, 1/1600 or 1/3200 was added and incubated for 1hr at 37°C. Following six washes, wells were incubated in the dark with biotinylated total IgG (Jackson ImmunoResearch) for 1hr at room temperature. Unbound antibodies were washed off (six times) prior to incubating wells in the dark with streptavidin HRP (BD pharmagen) for 30 minutes at room temperature. Wells were washed six times prior to development (100**μ**l, OPD; Sigma-Aldrich) for five minutes in the dark before termination with an equal volume of 1M HCl. Absorbance was determined at 492nm using a Biotek synergy H4 ELISA plate reader (Biotek, USA). Data were analysed using Gen5 software (version 2) and GraphPad Prism (version 6).

### Administration of TLR agonists and assessment of serum cytokine levels

TLR4 agonist, Lipopolysaccharide (LPS) (Sigma-Aldrich, derived from E.coli O127:B8), and TLR9 agonist, CpG1826 (Sigma-Aldrich, TCCATGACGTTCCTGACGTT with phosphorothioate linkages) were diluted in 0.9% saline (Baxter). Mice received 75μg of either, 2 hours prior to infection in 200μl volumes via intraperitoneal (*i*.*p*.) injection using 26G needles. To confirm efficacy of TLR agonists, serum cytokine concentrations at 9 hours post-treatment were assessed using Cytometric Bead Arrays (BD Bioscience) according to manufacturers’ instructions. Samples were acquired on a LSRII Fortessa analyser (BD Biosciences) and analysed using FCAP Array Version 3 (BD Biosciences) software.

### Assessment of immune-serum IgG binding under in vitro and in vivo conditions

To assess binding of IgG within immune serum to pRBC *in vivo*, blood from immune serum (or control non-immune serum) recipient mice was diluted (RPMI, 5 U/ml heparin sulphate) and for positive controls only, incubated with an anti-mouse RBC, mouse IgG2a antibody, clone 34-3C (1.5μg/ml; Abcam, Cambridge, MA, USA). RBC were washed three times in cold FACS buffer (1% w/v BSA, 5mM EDTA in PBS) prior to incubation on ice with detection reagent, FITC-conjugated goat anti-mouse IgG (3μg/ml; Biolegend, San Diego, CA). RBC were again washed three times, prior to staining with cell-permeant RNA/DNA stain, Syto84 (5 μM; Life Technologies) and DNA stain, Hoechst 33342 (10 μg/ml; Sigma), followed by immediate flow cytometric analysis on an LSRII Fortessa analyser (BD Biosciences) and FlowJo software (Treestar, CA, USA).

To assess *in vitro* immune serum IgG binding to pRBC, the same protocol was employed, except that RBC were first incubated *in vitro* with immune serum diluted in RPMI, and then stained as above. To further investigate IgG specificity for parasites, pRBC from infected mice were first fixed and permeabilised to expose parasite structures to antibodies (BD Cytofix/Cytoperm; BD Life Sciences), and were then stained and analysed as above with the exception that reagents were prepared in Cytofix/Cytoperm Wash buffer.

### Assessment of IgG binding via immunofluorescence microscopy

Immunofluorescence assays were performed as previously described [46]. Briefly, thin blood smears were made on glass slides, air dried and fixed in ice cold Acetone/Methanol (50:50) for 10 minutes. The slides were removed from the fixative and air dried prior to performing the assay. The slides were washed three times in 1X PBS and blocked in 3% BSA/1XPBS for 1 hour. The blocking solution was removed and the pooled non immune and immune sera added to the slides and incubated at room temperature for 2hrs (1:5dilution in 3%BSA/1XPBS). Rabbit anti-GAPDH antibodies were used as a counterstain (1:2000). The slides were washed 3 times in 1X PBS before addition of the secondary antibodies. Goat anti-mouse Alexa Fluor 488 and anti-rabbit 568 (1:400) secondary antibodies were prepared in 3%BSA/1XPBS and incubated for 1 h at room temperature. Slides were washed as described above and the parasite nuclei were stained with DAPI (2 μg/mL) for 10 min at room temperature. The slides were again washed prior to the mounting in p-phenylenediamine antifade and addition of a coverslip. Imaging was performed on a DeltaVision Elite Restorative Widefield Deconvolution Imaging System (GE Healthcare) using the oil immersion 100x UPLS Apo (1.4NA) objective. Samples were excited with solid state illumination (Insight SSI, Lumencor). The following filter sets with excitation and emission wavelengths were used: DAPI Ex390/18, Em435/48; FITC, Ex475/28, Em523/26; TRITC, Ex542/27, Em594/45; Cy5 Ex 632/22, 676/34 nm. Identical exposure settings were used for the non-immune and immune sera groups. Images were processed using the Fiji ImageJ software.

### Phagocyte and complement C3 depletion in vivo

Host phagocytes were depleted *in vivo* as previously described [29], with a single *i*.*v*. injection (via a lateral tail vein using 26G needles) of 200μl of clodronate-containing liposomes (www.clodronateliposomes.com) 2 days prior to passive transfer of serum. To deplete mice of the complement component, C3, 10μg of Cobra Venom Factor (CVF)(Quidel) was injected intraperitoneally 16 hr prior to infection (day -1), and then daily on days +1, +2 & +3; control mice were injected with saline. To evaluate the efficacy of CVF treatment, C3-depletion efficacy was measured in serum by sandwich ELISA. Costar EIA/RIA 96-well flat bottom plates were coated overnight at 4°C with 2.0μg/ml primary antibody (Goat IgG Fraction to Mouse Complement C3, MP Biomedicals) in PBS. Wells were washed three times (all washes in 0.005% Tween in PBS) and then blocked for 1 hr at room temperature (RT) with 1% BSA in PBS. Wells were washed three times, and 100μl of sera in duplicate at 1/9000 and 1/27000 was added and incubated for 1 hr at RT. Following six washes, wells were incubated in the dark with a secondary antibody (Peroxidase-Conjugated Goat IgG Fraction to Mouse Complement C3, MP Biomedicals) for 1hr at RT. Unbound antibodies were washed off six times prior to incubating wells in the dark with streptavidin HRP (BD Biosciences) for 30 mins at RT. Wells were washed six times prior to development with substrate (OPD; Sigma-Aldrich) for five minutes in the dark before termination with 1M HCl. Absorbance was determined at 492nm using Biotek synergy H4 ELISA plate reader (Biotek, USA).

### Flow cytometric assessment of cellular depletion in the spleen

To assess host cell depletion, 200μl of clodronate containing liposomes (www.clodronateliposomes.com) was administered to mice by a single *i*.*v*. injection (via a lateral tail vein using 26G needles), or control untreated, 3 days prior to assessment by flow cytometry. For flow cytometry analysis, spleens were collected, chopped, collagenase/DNase treated (0.1% (1mg/ml) Collagenase D and 0.05% (0.5mg/ml) DNase in RPMI 1640 at room temperature for 30 minutes with constant mixing, then homogenized through a 100μm cell strainer to create a single cell suspension. RBC were lysed by incubating samples with RBC lysing buffer (Sigma-Aldrich) for 5 minutes at RT and splenocytes were washed in FACS buffer. Cells were assessed for viability by staining with LIVE/DEAD Fixable Aqua Dead Cell stain kit (Life Technologies), according to the manufacturers’ protocol. Cells were FcγR-blocked (αCD16/32 (clone 93)), and stained for surface markers by incubating with the following antibodies on ice for 20 minutes: B220-PB (RA3-6B2), Ly6G-APCCy7 (1A8), MHC-II-PE (M5/114.15.2), CD11b-PercpCy5.5 (M1/70), CD11c-APC (N418), Ly6c-FITC (HK1.4), F4/80-PeCy7 (BM8). Samples were fixed in 2% paraformaldehyde and acquired on a BD LSR Fortessa Cell Analyser (BD Biosciences). Data were analysed using FlowJo software (Tree Star).

### Calculation of PMR and statistical analysis

To determine Parasite Multiplication Rate (PMR) for those parasites transitioning from Gen_0_ to Gen_1_ RBC, which also indicates the average number of new pRBC produced from each injected CTFR+ Gen_0_ pRBC over the first replication cycle *in vivo*, we calculated: [Gen_1_ pRBC at 25h]/[Gen_0_ pRBC at 1h]. Group means and standard error of the mean are presented in the text.

All comparisons of group means were performed using a one-way ANOVA followed by a post-hoc contrast analysis, unless otherwise stated (using the anova1.m and multcompare.m functions in MATLAB R2015b (8.6.0.267246)). Analysis of interactions between groups were assessed using a two-way ANOVA analysis (using the anovan.m function in MATLAB R2015b (8.6.0.267246)). Results from flow cytometric analysis were plotted in figures using GraphPad Prism (v7.02), with statistical comparisons between two groups at a single timepoint performed using non-parametric, Mann-Whitney tests unless stated otherwise.

## Supporting information

S1 Fig Generation of *Py*17XNL-immune serum, and its capacity to restrict parasite growth in ongoing *Py*17XNL-infection. (A) Immune mice were generated following blood-stage infection with *P*. *yoelli* 17XNL (Py17XNL). Infection was confirmed 12 *dpi*, and *Py*17XNL-specific IgG was measured 60–70 *dpi* in immune and age-matched non-immune mice (n = 5-30/group); data representative of more than five independent experiments, each with similar results. (B) Total parasitemia of individual *Py*17XNL-infected mice therapeutically treated at 3 *dpi* with a single dose of *Py*17XNL-immune or non-immune control serum (n = 6/group) and tracked for 36 hours thereafter; experiment conducted once.
(TIF)

S2 Fig Time course assessment of total parasitemia in mice treated with *Py*17XNL-immune and non-immune serum as well as in *Py*17XNL-immune and non-immune mice re-challenged with *Py*17XNL. Data shows alternative depiction of data from Figs 1 and 2, in which total parasitemia, regardless of CTFR status, was assessed in immune and non-immune serum treated mice as well as immune and non-immune mice.
(TIF)

S3 Fig *In vivo* maturation of Gen0 *Py*17XNL-infected pRBC is unaffected by exposure to *Py*17XNL-immune serum. Representative FACS plots gated on Gen_0_ (CTFR^+^) RBC in mice (n = 5/group) at indicated times after transfer into mice that had received either *Py*17XNL-immune or non-immune control serum 24h previously, showing parasite life-stages based on Hoechst and Syto84 profiles. Graphs show percentage of Gen_0_ pRBC in each life stage. Data are representative of >5 independent experiments, each showing similar results.
(TIF)

S4 Fig Gen0 pRBC from *rag1^-/-^* passage mice exhibit similar in vivo kinetics to Gen0 pRBC from WT passage mice. Using WT or *rag1*^*-/-*^ *Py*17XNL-infected passage mice for generating CTFR-labelled RBC, data indicate the loss of Gen_0_ (CTFR^+^) pRBC, and emergence over the first 3 days of Gen_1+_ (CTFR^-^) pRBC (shown on linear and logarithmic y-axes) in mice (n = 5/group) administered *Py*17XNL immune serum, or control non-immune serum. Data are representative of two independent experiments.
(TIF)

S5 Fig Purified IgG from *Py*17XNL-immune serum performs similarly to immune serum *in vivo*. (A) Coomassie stained 10% SDS PAGE gel showing serum, column flow-through (FT) and purified IgG eluate (10μg) from Protein G purification from non-immune and immune mice. Rat monoclonal IgG against mouse CD3 (Commercial Ab) was included as a control. (B) Mice (n = 5/group) were administered 500μg of *Py*17XNL immune purified IgG, control non-immune purified IgG, or non-purified serum from the same batch, 24h prior to receiving CTFR^+^ Gen_0_ pRBC containing *Py*17XNL, with Gen_0_ and Gen_1+_ pRBC monitored at times indicated.
(TIF)

S6 Fig Modifications to the immune serum passive transfer protocol do not influence pRBC clearance rates during *Py*17XNL challenge. (A) Mice (n = 5/group) were administered 150μl of *Py*17XNL immune serum, or control non-immune serum 2h prior to receiving CTFR^+^ Gen_0_ pRBC containing *Py*17XNL, with Gen_0_ and Gen_1+_ pRBC monitored at time points indicated. Experiment conducted once. (B). Mice (n = 3/group) were administered 400μl of *Py*17XNL immune serum, or control non-immune serum, or no serum (to examine non-specific effects of non-immune serum) 2h prior to receiving CTFR^+^ Gen_0_ pRBC containing *Py*17XNL, with Gen_0_ and Gen_1+_ pRBC monitored at time points indicated. Experiment conducted once.
(TIF)

S7 Fig Clodronate liposome treatment depletes splenic F4/80^+^ macrophages and CD11c^hi^ conventional dendritic cells. (A) FACs gating strategy employed to identify conventional dendritic cells (cDC; CD11c^hi^ MHC-II^+^), macrophages (mΦ; F4/80^+^ CD11b^-^), neutrophils (Ly6G^+^CD11b^lo^) and monocytes (CD11b^hi^Ly6G^-^). (B-D) Mice were administered 200μl of clodronate-containing liposomes via *i*.*v*. injection, or control untreated, three days prior to assessment by flow-cytometry. Representative FACs plots are shown comparing the percentages of (B) cDCs, (C) macrophages, (D) neutrophils and monocytes in the spleen. Graphs show the total cell counts per spleen for these cells, with fold-reductions in each cell-type shown. Each dot represents an individual mouse. Statistics: Mann-Whitney t-test, **P < 0.01; *P<0.05.
(TIF)

S8 Fig *Py*17XNL-specific antibodies do not require complement-mediated direct killing for *in vivo* function. (A) Schematic showing that 4 groups of mice (n = 5/group) were treated with immune or non-immune control serum, treated with cobra venom factor (CVF) or control saline indicated by green arrows, and challenged with CTFR^+^ *Py*17XNL-infected pRBC. (B) Serum C3 levels assessed at various times post-challenge (n = 5/group). (C) loss of Gen_0_ (CTFR^+^) pRBC and (D) emergence over the first 25 hours of Gen_1+_ (CTFR^-^) pRBC in C3-replete, or CVF-treated mice receiving immune or non-immune control serum, with later timepoints beyond 25 hours also shown. Data representative of two independent experiments showing similar results.
(TIF)
