# Supplementary figures and images for: Plasmodium-specific antibodies block in vivo parasite growth without clearing infected red blood cells
Source: PLoS Pathog. 2019 Feb 27;15(2):e1007599. doi: 10.1371/journal.ppat.1007599 (PMC6411214; doi:10.1371/journal.ppat.1007599)

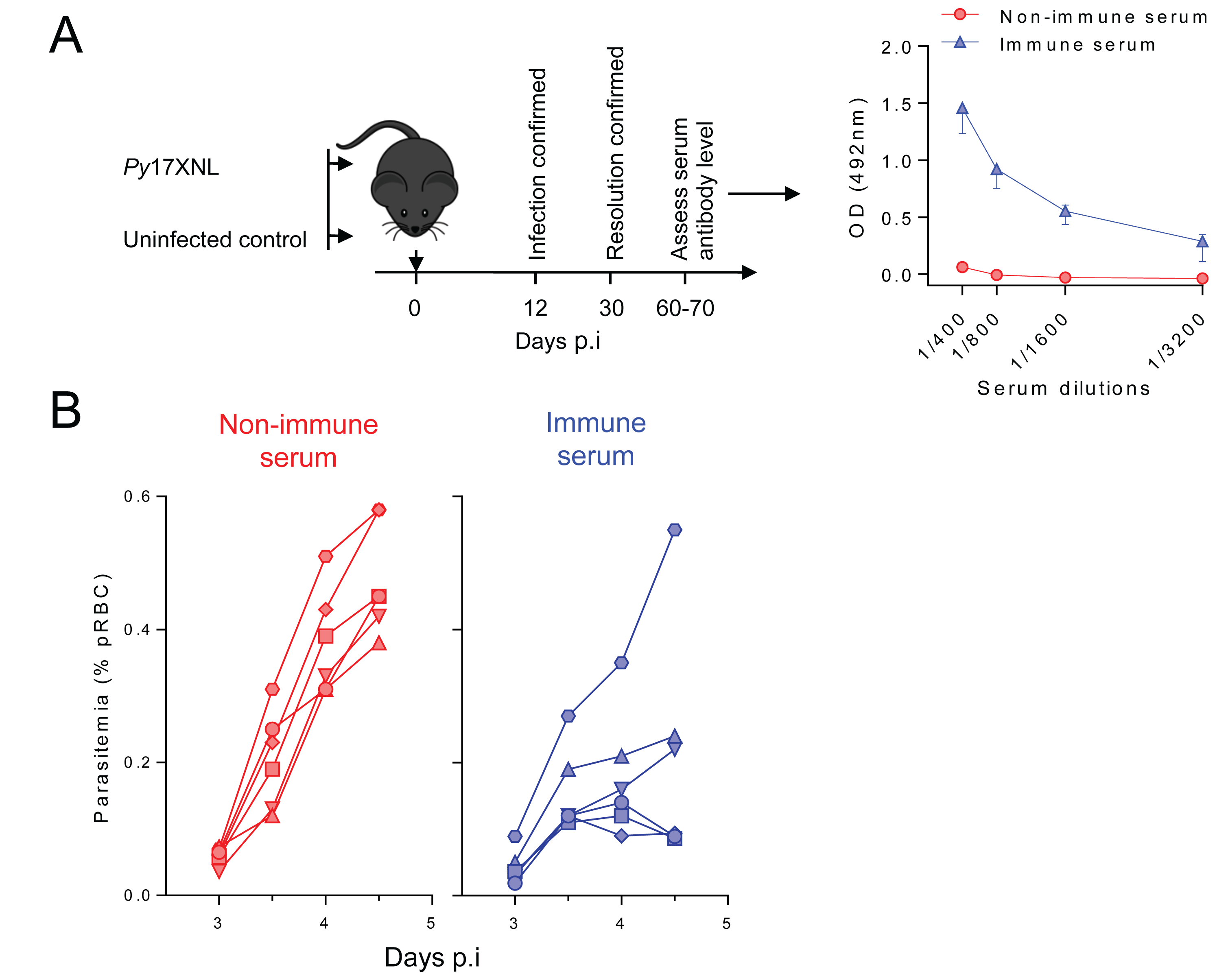

Supplement: S1 Fig — (A) Immune mice were generated following blood-stage infection with P. yoelli 17XNL (Py17XNL). Infection was confirmed 12 dpi, and Py17XNL-specific IgG was measured 60–70 dpi in immune and age-matched non-immune mice (n = 5-30/group); data representative of more than five independent experiments, each with similar results. (B) Total parasitemia of individual Py17XNL-infected mice therapeutically treated at 3 dpi with a single dose of Py17XNL-immune or non-immune control serum (n = 6/group) and tracked for 36 hours thereafter; experiment conducted once. (TIF) [file ppat.1007599.s001.tif]

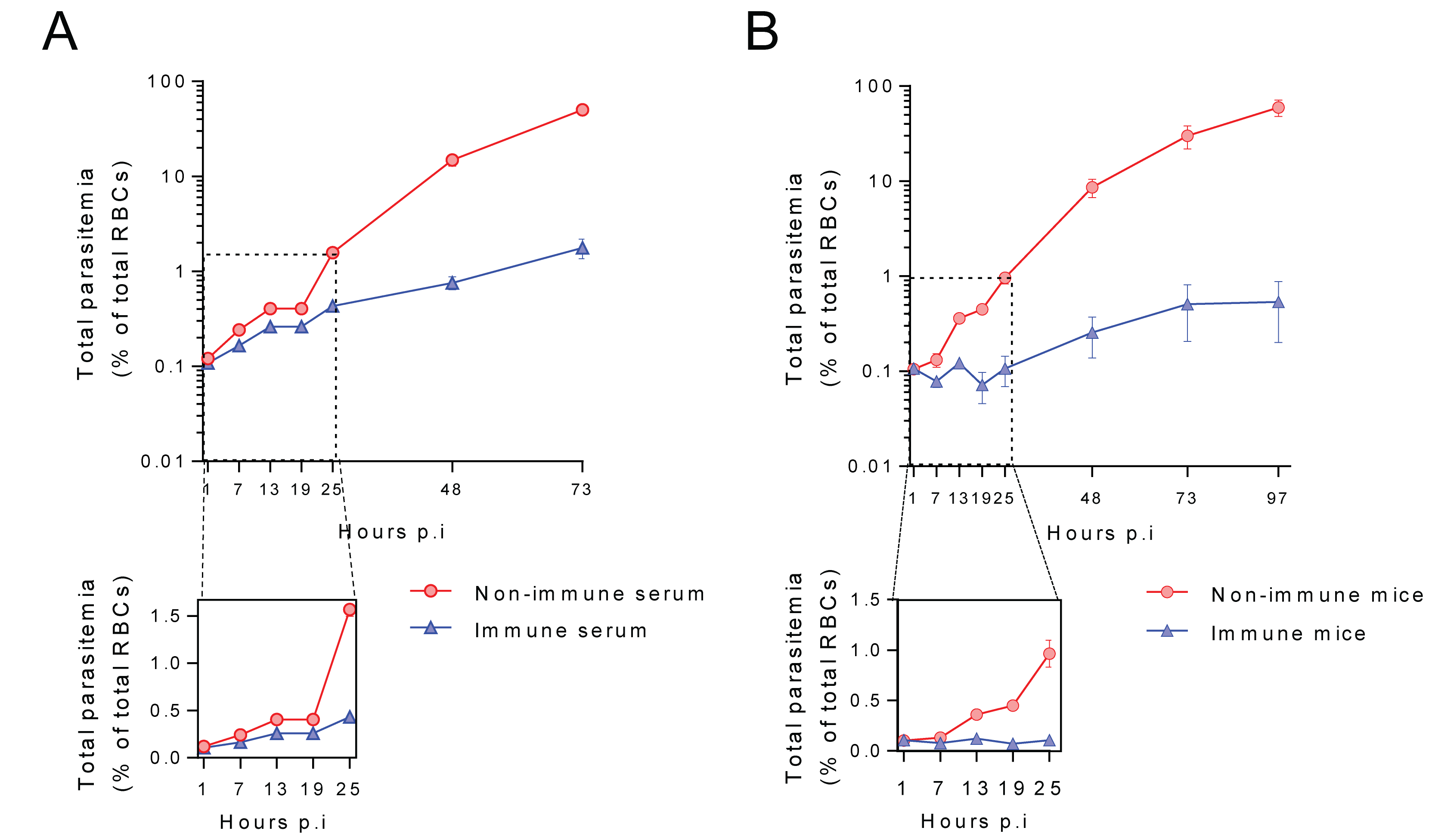

Supplement: S2 Fig — Data shows alternative depiction of data from Figs 1 and 2, in which total parasitemia, regardless of CTFR status, was assessed in immune and non-immune serum treated mice as well as immune and non-immune mice. (TIF) [file ppat.1007599.s002.tif]

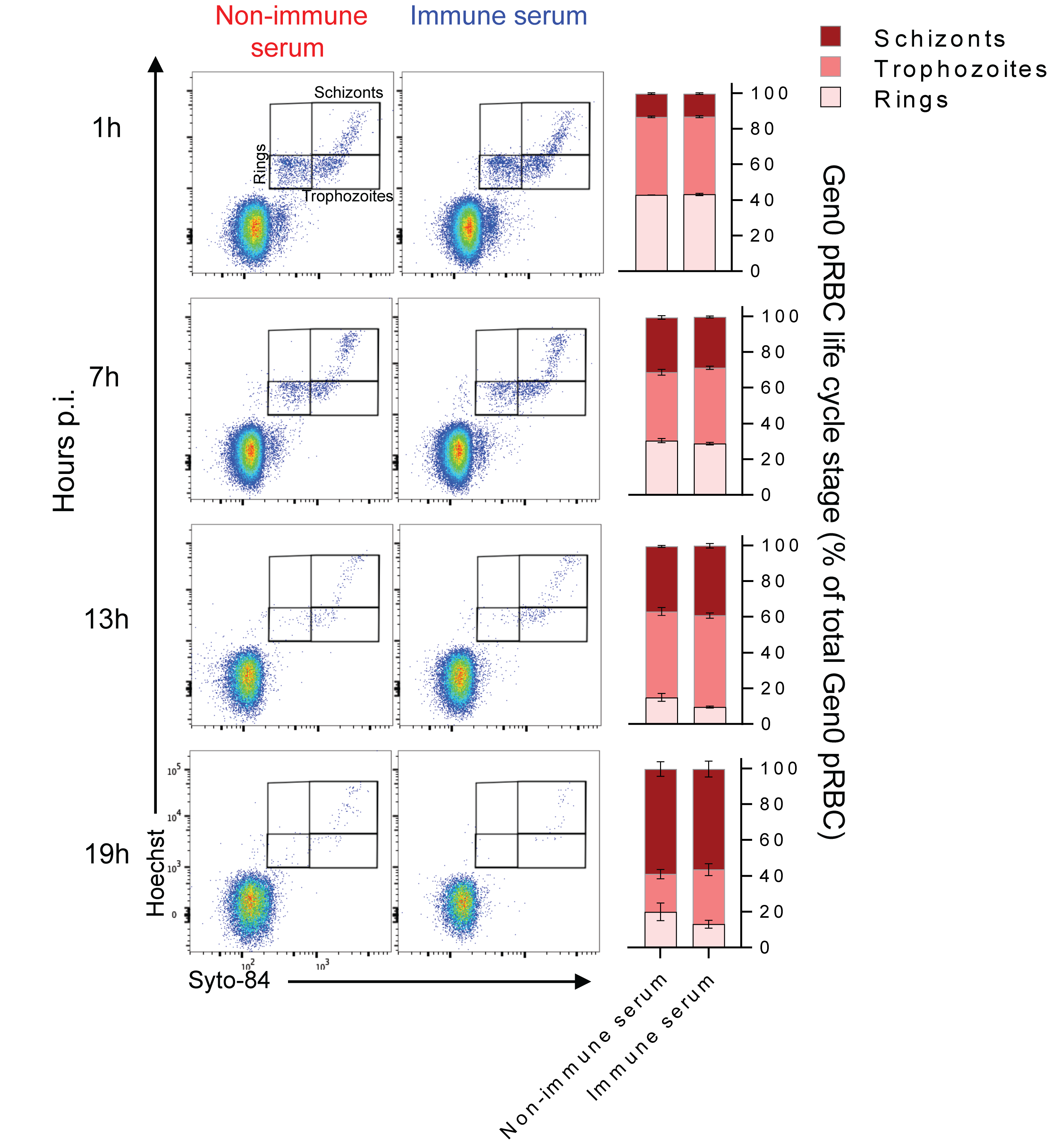

Supplement: S3 Fig — Representative FACS plots gated on Gen0 (CTFR+) RBC in mice (n = 5/group) at indicated times after transfer into mice that had received either Py17XNL-immune or non-immune control serum 24h previously, showing parasite life-stages based on Hoechst and Syto84 profiles. Graphs show percentage of Gen0 pRBC in each life stage. Data are representative of >5 independent experiments, each showing similar results. (TIF) [file ppat.1007599.s003.tif]

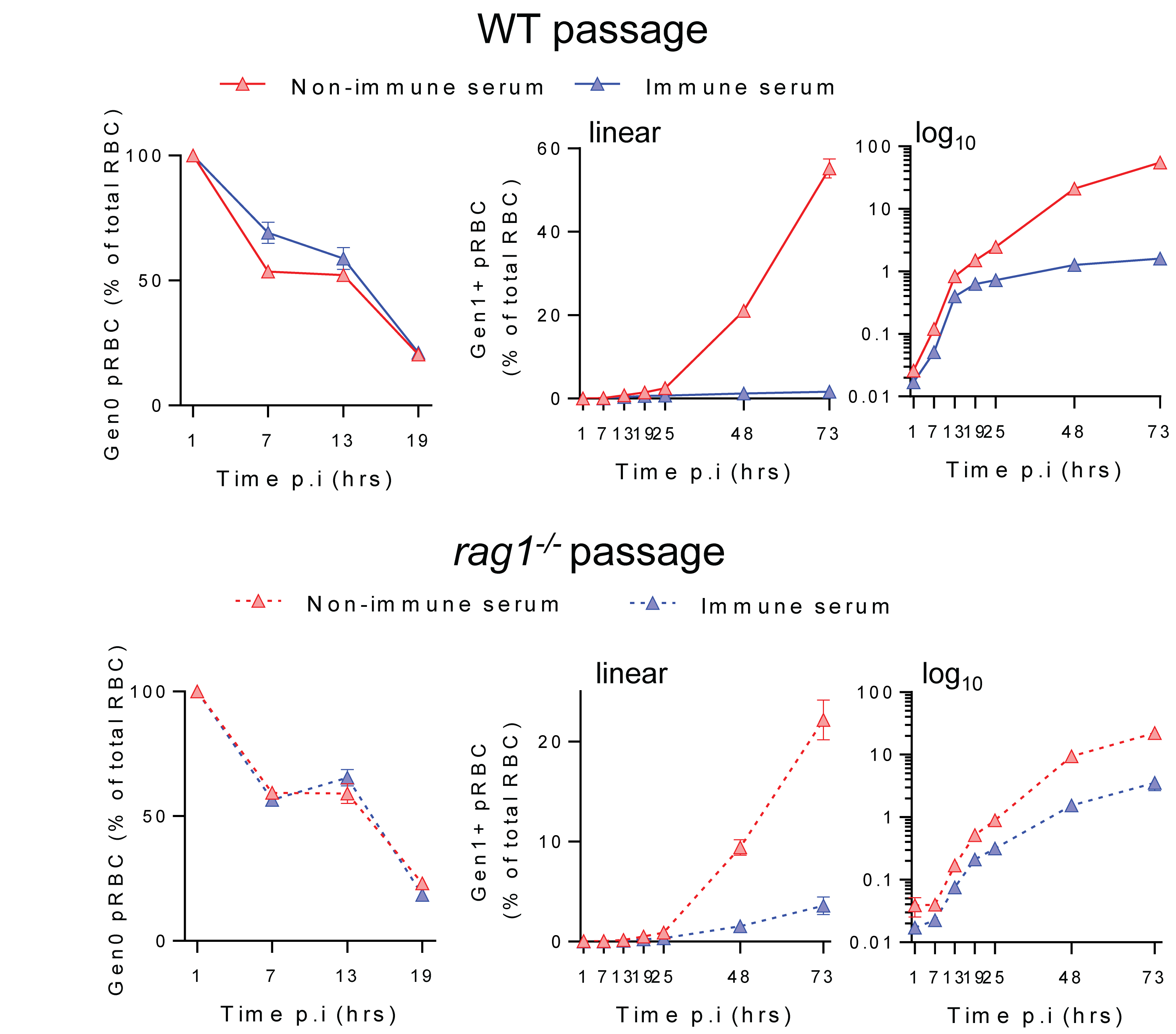

Supplement: S4 Fig — Using WT or rag1-/- Py17XNL-infected passage mice for generating CTFR-labelled RBC, data indicate the loss of Gen0 (CTFR+) pRBC, and emergence over the first 3 days of Gen1+ (CTFR-) pRBC (shown on linear and logarithmic y-axes) in mice (n = 5/group) administered Py17XNL immune serum, or control non-immune serum. Data are representative of two independent experiments. (TIF) [file ppat.1007599.s004.tif]

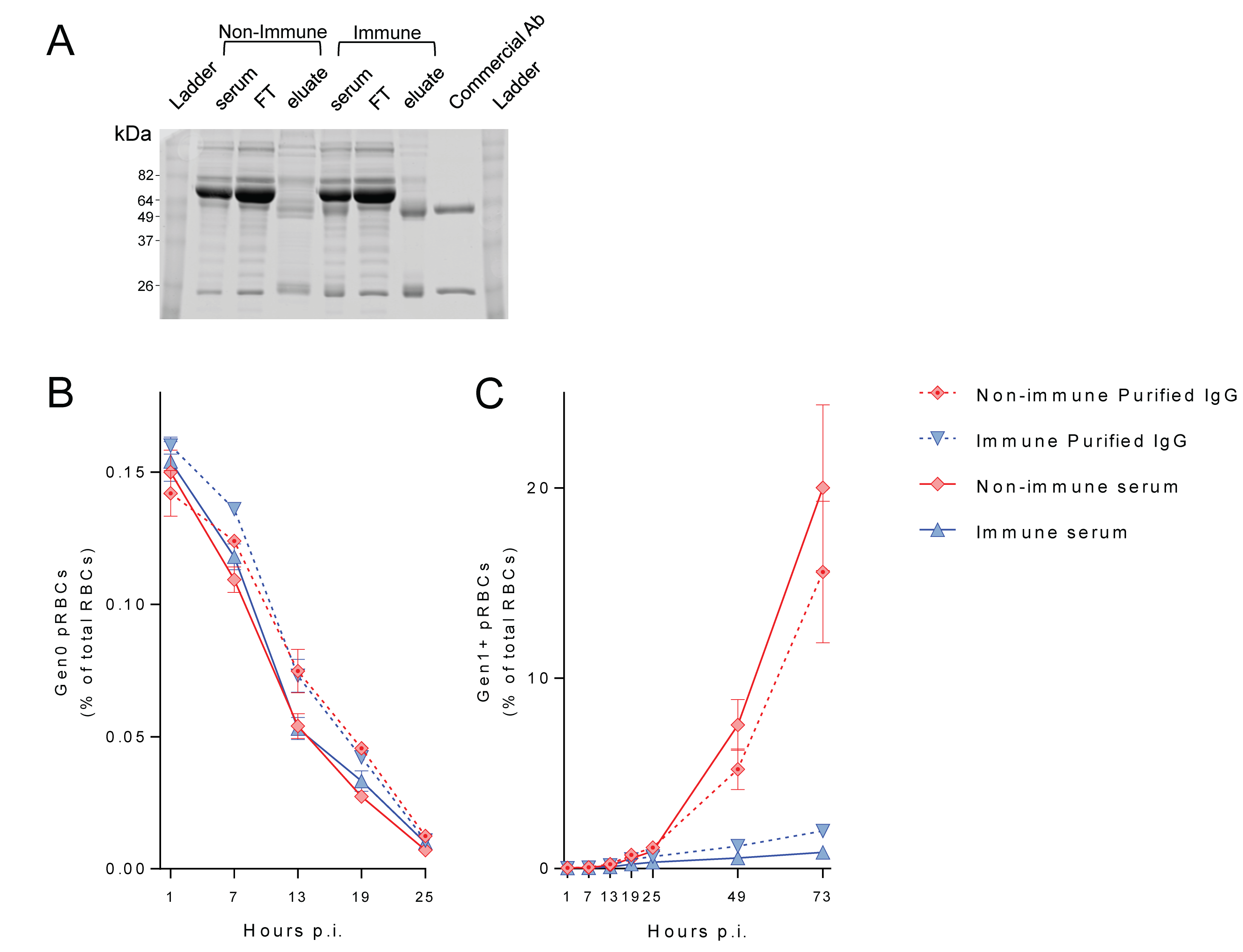

Supplement: S5 Fig — (A) Coomassie stained 10% SDS PAGE gel showing serum, column flow-through (FT) and purified IgG eluate (10μg) from Protein G purification from non-immune and immune mice. Rat monoclonal IgG against mouse CD3 (Commercial Ab) was included as a control. (B) Mice (n = 5/group) were administered 500μg of Py17XNL immune purified IgG, control non-immune purified IgG, or non-purified serum from the same batch, 24h prior to receiving CTFR+ Gen0 pRBC containing Py17XNL, with Gen0 and Gen1+ pRBC monitored at times indicated. (TIF) [file ppat.1007599.s005.tif]

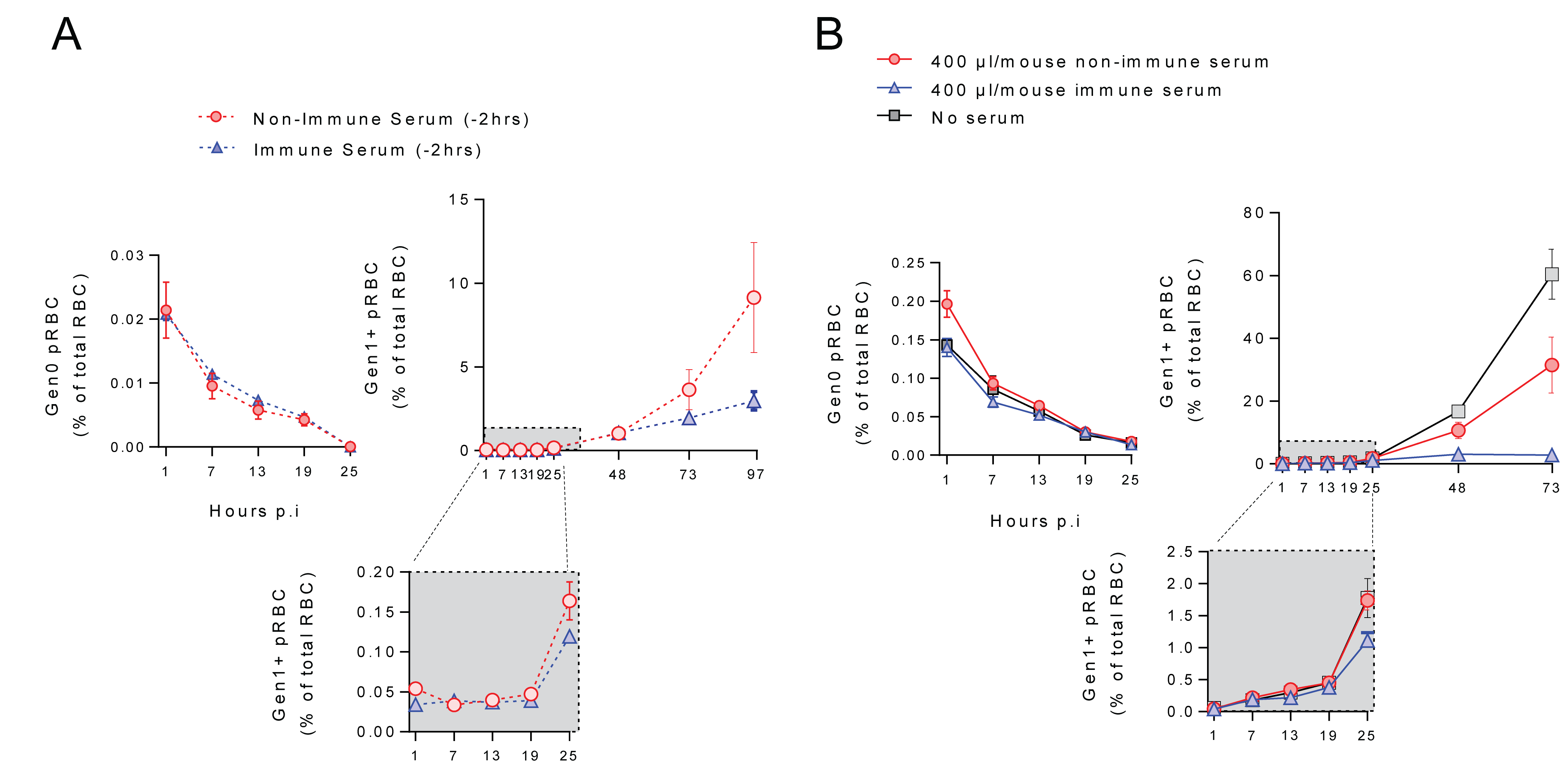

Supplement: S6 Fig — (A) Mice (n = 5/group) were administered 150μl of Py17XNL immune serum, or control non-immune serum 2h prior to receiving CTFR+ Gen0 pRBC containing Py17XNL, with Gen0 and Gen1+ pRBC monitored at time points indicated. Experiment conducted once. (B). Mice (n = 3/group) were administered 400μl of Py17XNL immune serum, or control non-immune serum, or no serum (to examine non-specific effects of non-immune serum) 2h prior to receiving CTFR+ Gen0 pRBC containing Py17XNL, with Gen0 and Gen1+ pRBC monitored at time points indicated. Experiment conducted once. (TIF) [file ppat.1007599.s006.tif]

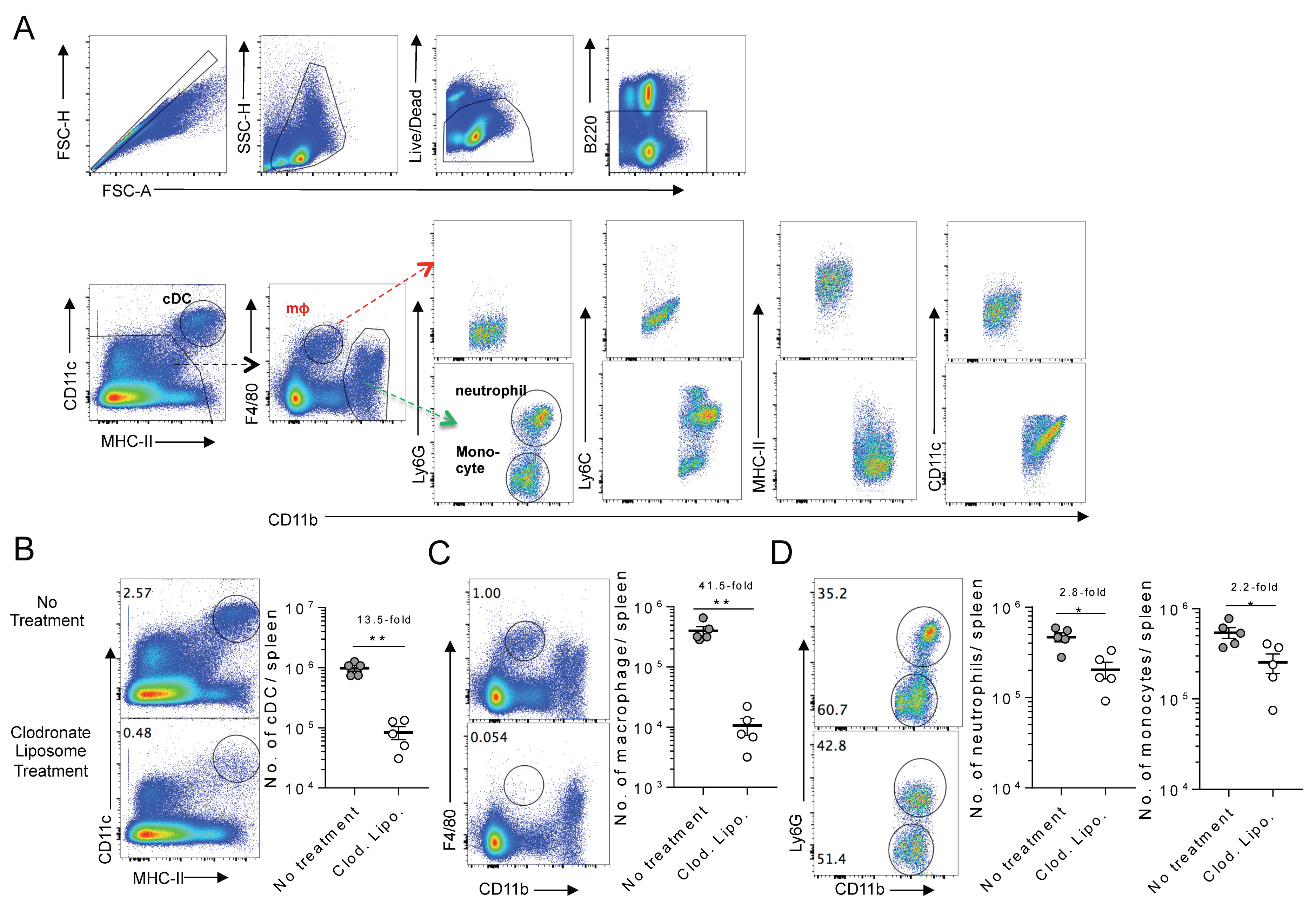

Supplement: S7 Fig — (A) FACs gating strategy employed to identify conventional dendritic cells (cDC; CD11chi MHC-II+), macrophages (mΦ; F4/80+ CD11b-), neutrophils (Ly6G+CD11blo) and monocytes (CD11bhiLy6G-). (B-D) Mice were administered 200μl of clodronate-containing liposomes via i.v. injection, or control untreated, three days prior to assessment by flow-cytometry. Representative FACs plots are shown comparing the percentages of (B) cDCs, (C) macrophages, (D) neutrophils and monocytes in the spleen. Graphs show the total cell counts per spleen for these cells, with fold-reductions in each cell-type shown. Each dot represents an individual mouse. Statistics: Mann-Whitney t-test, **P < 0.01; *P<0.05. (TIF) [file ppat.1007599.s007.tif]

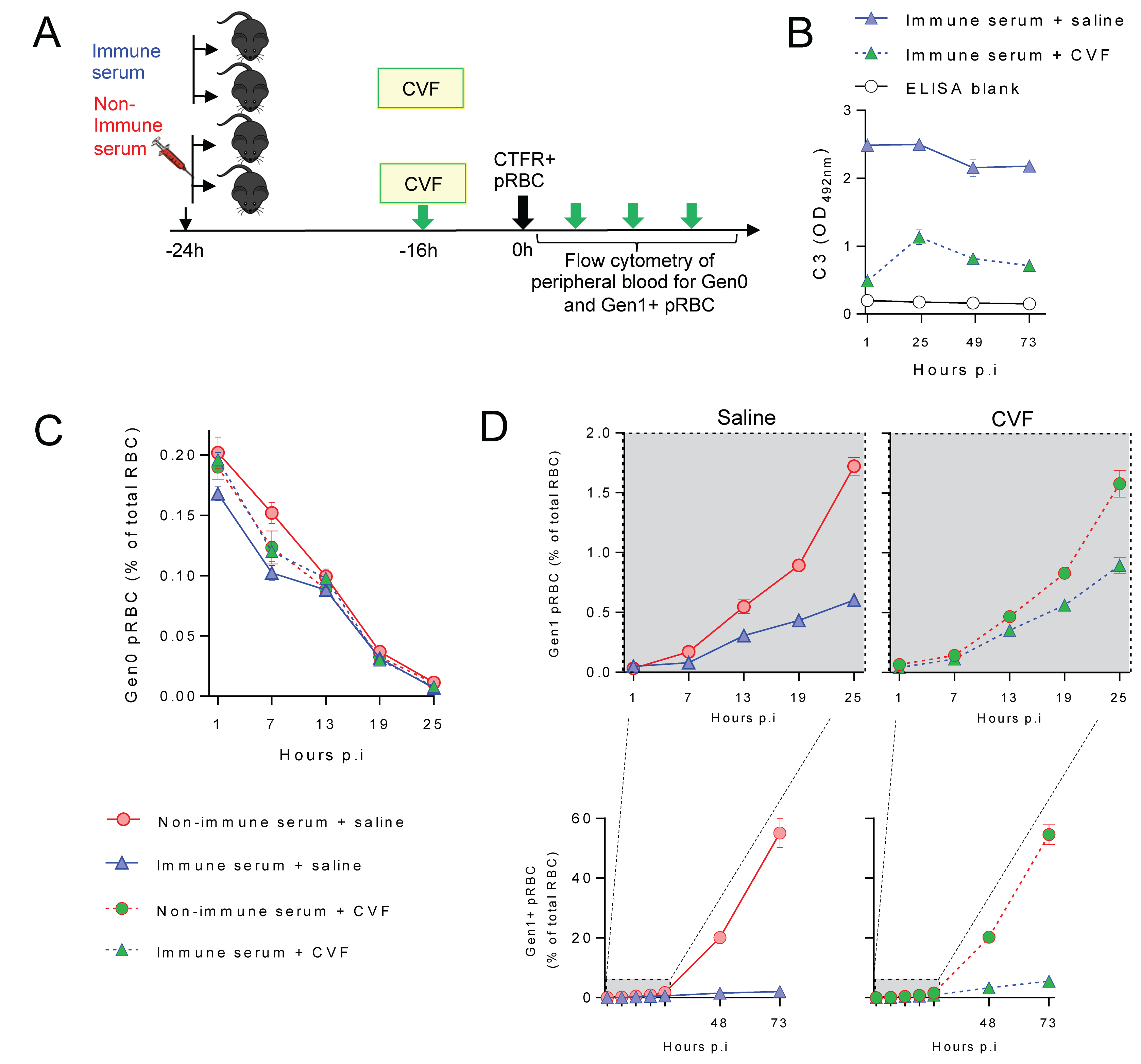

Supplement: S8 Fig — (A) Schematic showing that 4 groups of mice (n = 5/group) were treated with immune or non-immune control serum, treated with cobra venom factor (CVF) or control saline indicated by green arrows, and challenged with CTFR+ Py17XNL-infected pRBC. (B) Serum C3 levels assessed at various times post-challenge (n = 5/group). (C) loss of Gen0 (CTFR+) pRBC and (D) emergence over the first 25 hours of Gen1+ (CTFR-) pRBC in C3-replete, or CVF-treated mice receiving immune or non-immune control serum, with later timepoints beyond 25 hours also shown. Data representative of two independent experiments showing similar results. (TIF) [file ppat.1007599.s008.tif]
